# Use it or lose it: A model-based assessment of the hypothesis that European Neanderthals relied on wildfires to create their campfires

**DOI:** 10.12688/openreseurope.20477.1

**Published:** 2025-07-30

**Authors:** Andreu Arinyo i Prats, Dennis Sandgathe, Felix Riede, Mark Collard

**Affiliations:** 1 Department of Archaeology and Heritage, Aarhus University, Højbjerg, Moesgård Allé 20, Central Denmark Region, 8270, Denmark; 2 Laboratory of Human Evolutionary Studies, Department of Archaeology, Simon Fraser University, 8888 University Drive Burnaby, British Columbia, B.C. Canada V5A 1S6, Canada; 3 Human Evolution Behavior and Culture, Max Planck Institute for Evolutionary Anthropology, Deutscher Platz 6, Leipzig, 04103, Germany

**Keywords:** fire use, cultural evolution, cultural loss, Neanderthals, climate, modeling, archaeology

## Abstract

**Background:**

There remains debate about the pyrotechnical capabilities of the Neanderthals. Evidence of fire has been found at many Middle Palaeolithic sites, widely accepted to be associated with Neanderthals. However, multiple Neanderthal sites show a marked decrease in evidence for fire use during colder periods. This counterintuitive pattern was explained by the possibility that some Neanderthal groups were unable to create fire at will and relied on wildfire. Here, we evaluate the plausibility of this ‘wildfire hypothesis’ through formal modeling.

**Methods:**

We computed the probability of a group of Neanderthals losing campfire-making skills due to cultural loss. The EMBERS model codes four empirically motivated mechanisms of skill loss: variability in use, period in between uses, memory decay and number of experts.

**Results:**

Our results indicate that losing the ability to use wildfire was more likely than retaining the it for most of our parameter values within reasonable ranges. Significantly, demography, in the form of expert numbers, was the least significant mechanism of loss. The rate of memory loss at group level, and intervals between uses where significantly more important than demography. Large variability in use was, by far, the strongest driver of loss. These results, linked with the estimated climatic, mnemonic, and demographic conditions for the Neanderthal's settlements in the glacial periods, support the plausibility of that the wildfire hypothesis and highlights the need to pay more attention to cultural loss as a factor in cultural evolution.

**Teaser:**

Our modeling shows that cultural loss can trigger the loss of campfire-use by some Neanderthal Groups in the context of cold climatic transition.

## Introduction

The development of the ability to control and create fire is generally accepted to have been a key event in human evolution ^1, 2^. Many benefits of these abilities have been recognized. In addition to the obvious ones of providing heat and light and enabling food to be cooked, fire would have allowed individuals to manufacture a range of entirely new artefacts, protect themselves against pests and predators, and increase hunting returns via landscape burning ^3– 5^. The advantages of fire are so great, according to some researchers, that its use influenced the evolution of our digestive and nervous systems ^6, 7^. It has even been suggested that we, modern humans, are obligate fire users ( *i.e.*, we cannot survive without fire) ^7^. By the same token, however, ethnographic evidence amply illustrates how difficult fire is to control and curate: Even for late Holocene *Homo sapiens* with elaborate cultural scaffolds for high-fidelity transmission of knowledge and know-how across generations, the ability to create and safely transport embers has repeatedly been lost ^8, 9^.

While there is consensus that the development of the ability to create and control fire was a milestone in human evolution, several issues concerning the history of pyrotechnology remain poorly understood. It is unclear, for example, how early hominins managed to occupy northern Europe without fire ^10^. Another poorly understood issue—and the one on which we focus in this paper—is whether our closest relatives, the Neanderthals ( *Homo neanderthalensis*), were consistently able to create fire at will and maintain this ability via social transmission across the many millennia of their tenure. Evidence of fire has been found at many European Middle Palaeolithic sites, which means there is no question that Neanderthals used fire. Yet, opinions differ as to whether they were able to create fire from scratch. Many researchers have assumed that the Middle Palaeolithic fire evidence indicates that Neanderthals were readily capable of creating, controlling, and curating fire ^11, 12^. However, over the last 15 years the notion that all Neanderthals at all times were fully capable pyrotechnologists has been challenged and an alternative possibility proposed, which is that some, perhaps many, Neanderthal groups relied on wildfire to start their campfires and, as a consequence, were prone to losing the know-how required to manage fire during periods of climatic cooling, when wildfires were less frequent ^13– 16^.

Sandgathe and colleagues ^13– 16^ developed this hypothesis to explain results of analyses of fire residues at two caves in southwest France that were occupied by Neanderthals, Pech de l’Azé IV and Roc de Marsal. Sandgathe and colleagues ^13– 16^ showed that the Neanderthals who occupied these sites frequently used fire when the climate was temperate but greatly reduced their use of it, or perhaps even stopped using it, as conditions became increasingly cold. Sandgathe and colleagues ^13, 14^ proposed that this counterintuitive pattern indicates that at least some groups of Neanderthals relied on naturally occurring fire to make their campfires. They argued that this hypothesis explains the significant decrease in the use of fire during cold climatic periods by Neanderthals at the two sites because lightning strikes are more common in temperate conditions than in cold ones and, therefore, so are wildfires.

The pattern of less evidence for fire use during colder periods than in warmer ones is not limited to Pech de l’Azé IV and Roc de Marsal. Sandgathe and colleagues have since documented it at a number of other sites. In 2018, they demonstrated that the pattern is also seen at a third Middle Palaeolithic site in southwest France, Combe Grenal. More recently, they showed that the pattern occurs at Middle Palaeolithic sites in other parts of Europe ^16^. In this study, they analyzed the percentage of burned lithics in layers deposited in more temperate conditions versus layers deposited in cooler conditions at seven Middle Palaeolithic sites and an Upper Palaeolithic site. Four of the Middle Palaeolithic sites—Abri du Maras, Abric Romani, Sesselfelsgrotte, and Kulna—are not in southwest France. Abri du Maras is in southeast France; Abric Romani is in Spain; Sesselfelsgrotte is in Germany; and Kulna is in the Czech Republic. Sandgathe and colleagues found that the percentage of burned lithics was higher in layers deposited in warmer conditions than in layers deposited in cooler conditions at all of the Middle Palaeolithic sites. This is consistent with their earlier results ^13, 14^ and suggests that the pattern of greatly reduced evidence for fire use during colder periods than in more temperate ones is a common pattern.

In the time since Sandgathe and colleagues ^13– 16^ first discussed their hypothesis in print, a number of researchers have put forward counter arguments. The most challenging of these relates to the plausibility of the hypothesis. Sorensen ^17^ argued that it is likely that there would only have been “modest differences in fire ignition frequencies between climatic periods” ( 16, pg. 19). The corollary of this, according to Sorensen ^17^, is that Neanderthals would still have encountered wildfires in the landscape in cold and dry periods, and therefore an inability to create fire and reliance on harvesting wildfire cannot explain the decline. Sandgathe and colleagues ^15^ rejected this criticism on the grounds that there is a near-universal consensus among climatologists, ecologists, and atmospheric scientists that lightning-caused wildfires are much more common in warm and humid conditions than in cold and dry ones. Although Sandgathe and colleagues ^15^ were correct about the consensus among specialists regarding the association between lightning-caused wildfires and climatic conditions, Sorensen’s point about Neanderthals still encountering wildfires in cold periods is well taken. Lightning strikes would not have stopped entirely as climatic conditions deteriorated. Rather, they would have become less frequent and more irregular. Thus, a key question regarding the plausibility of Sandgathe and colleagues’ ^13– 16^ hypothesis is, Could multiple groups of Neanderthals have lost the ability to use wildfire to create campfires as climatic conditions became colder and drier, even though they still encountered wildfires on occasion?

That hominins occupying the, at times, frigid mid-to-high latitudes of Europe could have lost so patently useful a skill as employing wildfire to light a campfire seems implausible. However, ethnographic evidence suggests that the loss of useful skills is actually a relatively common occurrence among humans, especially in groups that are small and isolated. For example, the loss of the ability to create two useful technologies in parts of Oceania prior to the arrival of Europeans was documented by Rivers ^18^. One of these technologies was the canoe. Rivers recounted that the people of the Torres Islands, had previously made canoes but were no longer able to do so, and that the same held for the people of the island of Mangareva. The other technology was the clay pot. By comparing the distribution of archaeologically recovered pottery sherds with ethnographic accounts of pottery manufacture, Rivers demonstrated that the number of islands on which clay pots were produced had decreased over time. Rivers argued that the absence of suitable raw materials could not explain all of these losses. Among the alternative factors Rivers argued should be considered are religious beliefs, interaction with immigrants, and the loss of communities of specialist craft producers due to catastrophes. Boyd *et al.* ^19^ highlighted another pertinent case that illustrates that skills can be lost even if they are useful—the Polar Inuit of northwest Greenland. When European explorers visited this group in the mid 19 ^th^ century, they found that they remembered kayaks, bows-and-arrows, leisters, and heat-saving igloo-entrances, but no longer knew how to make them. The Polar Inuit explained that the know-how required to produce these items had been lost due to an epidemic in the 1820s that killed the group’s elders, who were its most knowledgeable members. These ethnographic examples suggest it is plausible that some Neanderthals could have lost the ability to use wildfire to light campfires even though it was useful skill.

There are also a number of archaeological examples of the loss of useful skills. In view of the space constraints, we will highlight just two. The details of the first example were elucidated by Riede ^20^. Riede showed that towards the end of the Pleistocene some hunter-gatherer groups in Northern Europe stopped using bow-and-arrow technology in the aftermath of the massive Laacher See Eruption, which occurred around 13,000 BP. Riede attributed the disappearance of bow-and-arrow technology to the groups becoming isolated due to the ash fallout. The second example concerns concrete. Scholars have long been puzzled by the abandonment of the use of concrete in many parts of Europe after the collapse of the Roman Empire, given concrete’s clear advantages for construction ^21, 22^. These archaeological examples also suggest it is plausible that some Neanderthal groups could have lost the ability to use wildfire to light campfires even though it was useful skill.

Further support for the plausibility of Sandgathe and colleagues’ ^14^ hypothesis is provided by studies reported by McCauley *et al.* ^9^ and Sugiyama ^23^. McCauley *et al.* ^9^ consulted ethnographic texts for a sample of 93 hunter-gatherer groups and collected data pertaining to fire use in settlements. McCauley *et al.* ^9^ found that several groups did not know how to make fire at the time the ethnographic data were collected. The groups in question were the Onges, Yuquí, Warlpiri, Sirionó, and northern Aché. The Onges and Yuquí collected natural fire and then conserved it for as long as possible. If an individual’s fire went out, they borrowed a firebrand from a neighbor. The Warlpiri were entirely reliant on industrially produced matches to make fire. The Sirionó explained to ethnographers that they used to know how to create fire with a friction method but no longer possessed this knowledge. If all their fires were extinguished, the Sirionó raided nearby settlements for fire. Not all Aché groups were able to make fire with traditional methods at the time of contact in the 1970s. The Southern Aché were able to do so but the Northern Aché were no longer able to create fire with traditional methods, and they were only able to remember some of the details of the methods. Importantly, McCauley *et al.* ^9^ only recorded a practice as absent when the relevant ethnographic reports specifically stated that the group did not engage in the practice, so the absence of the ability to make fire in these cases is reliable. In her study, Sugiyama ^23^ analyzed a large sample of hunter-gatherer oral narratives pertaining to the acquisition of fire. Her results underscore that pyrotechnical knowledge was in fact highly variable among ethnographically documented hunter-gatherers. They also underscore that creating fire from scratch was not a trivial matter. This is indicated by the fact that fire was obtained by raiding neighboring groups in a number of the oral narratives, despite the obvious risks of raiding. Additionally, Sugiyama’ ^23^ study shows that all the stages of fire-making and fire-keeping were scaffolded by complex oral traditions. Together, McCauley *et al.*’s ^9^ and Sugiyama’s ^23^ findings indicate that, prior to the development of friction matches in the 19 ^th^ century CE, the know-how required to manage fire would have been much more fragile and easily lost than is usually assumed.

In the present paper, we report a study that was designed to shed further light on the plausibility of Sandgathe and colleagues’ ^13– 16^ hypothesis. In the study, we modeled a scenario in which a Neanderthal group solely used lightning-caused wildfire to create its campfires, passed on the relevant techniques via social learning, and was isolated from other Neanderthal groups for long periods of time, which is consistent with the results of recent analyses of ancient DNA derived from Neanderthal remains from the site of Grotte Mandrin in southern France ^24^. We call the model we developed ‘EMBERS’. EMBERS was designed to enable us to estimate the probability of a Neanderthal group retaining the ability to use wildfire to create campfires in the face of variation in the interval between occurrences of wildfire (e.g., once every two years *vs* once every five years) and the level of unpredictability associated with these intervals (e.g., once every two years with a 10% variability on the interval *vs* once every five years with a 50% variability on the interval).

Before proceeding further, it is important to clarify that neither Sandgathe and colleagues’ ^13– 16^ hypothesis nor our study assumes the existence of cognitive differences between Neanderthals and modern humans. Critics often argue that Sandgathe and colleagues’ ^13– 16^ hypothesis requires Neanderthals to have been cognitively inferior to modern humans. This is not the case, however. The putative inability of European Neanderthals to create fire from scratch could have been due to the nature of their cognition, but equally it could have been due to non-cognitive factors, in the same way that the failure of some modern human groups to invent certain technologies (e.g., the wheel, the bow-and-arrow) or—as noted above—to lose fire-making skills had nothing to do with their cognitive abilities and was instead a consequence of factors like environmental conditions, demography, and chance. In other words, Sandgathe and colleagues’ ^13– 16^ hypothesis is agnostic about why Neanderthals did not develop the ability to create fire at will. The criticism also does not hold for our study. We only included one cognition-related variable in our model—the time it takes to forget the skills required to use wildfire to create a campfire and then maintain it—and the values we used for this variable were derived from previously published work involving living people. Thus, we also did not assume the existence of cognitive differences between Neanderthals and modern humans. On the contrary, we assumed that Neanderthals were identical to modern humans with respect to the one aspect of cognition that we included in the study.

## Key assumptions of the model

Modeling past hominin behavior always involves making multiple assumptions ^25^. Some of these assumptions have substantial impacts on the results; others have only minor effects. In this section, we outline the assumptions of EMBERS that fall into the former category.

One of the main assumptions of EMBERS is that the package of knowledge, skills, and equipment that would have enabled the Neanderthal group to use wildfire to create a campfire is sufficiently complex that it cannot be reinvented from start to finish via individual learning and therefore must be the result of cumulative cultural evolution. The assumption can be justified, we believe, by considering the actions necessary to use wildfire to start a campfire in a temperate zone. These actions include ( *a*) locating a wildfire in the landscape; ( *b* ) identifying a suitable ember to collect; ( *c*) transporting the ember; ( *d*) identifying, collecting, drying, and storing kindling and firewood ^26^; ( *e*) deciding on a suitable location for a fire in the camp ( *i.e.*, a location that is sheltered from the wind and does not lead to heat and smoke affecting other communal activities) ^26– 28^; ( *f*) arranging the kindling and firewood into one of the several possible fire lays (e.g., teepee lay, lean-to lay) ^27^; ( *g*) adding firewood to the growing fire in such a manner that air can still circulate and prevent the build-up of carbon monoxide ^28, 29^. Each of these actions involves knowledge, skills, and in some cases, special equipment. For example, starting fire from an ember requires detailed knowledge about the properties of tinder and wood ^27^, while the transportation of an ember has to be carried out in such a way that the ember does not harm the person carrying it. It also has to be carried out in such a way that the ember is not extinguished before arrival at the camp. And these are not the only relevant actions. A group using wildfire to create a campfire likely will also try to maintain the fire for an extended period of time, and, if the group is like ethnographically-documented hunter-gatherers ^9^, they will also try to transport fire between camps. Again, these actions involve knowledge, skills, and in the case of transporting fire between camps, special equipment ^26, 27, 30, 31^. Given the number and complexity of the actions involved, and the fact that some of them have to be carried out in a particular order, it is, we contend, highly unlikely that the WCP can be learned in its entirety through individual learning. It almost certainly has to develop through repeated experimentation, evaluation, and the transmission of knowledge and skills between generations. That is, it almost certainly has to be the product of cumulative cultural evolution. In line with this, from now on, we will refer to the package of knowledge, skills, and equipment as the ‘Wildfire Cultural Package’ or the WCP for short.

Crucially for present purposes, knowledge, skills, and technology assembled by cumulative cultural evolution can deteriorate and even disappear ^19, 32– 34^. This can occur as a result of the loss of the relevant knowledge and skills (e.g., due to forgetting, destruction of books) and/or the loss of group members with the relevant knowledge and skills ( *e.g.*, due to death, migration, burning of manuscripts) ^35– 37^. When creating EMBERS, we opted to model cultural loss as the reduction in the number of experts. We defined an expert as an individual with the knowledge and skills necessary to use wildfire to create and curate campfires (i.e., someone capable of using the WCP). We assumed that the number of experts undergoes exponential decay when the WCP is not utilized and immediately returns to a fixed maximum level each time the WCP is employed, providing that the number of experts does not drop below one during the interval of non-use.

Few longitudinal studies of the loss of knowledge and skills have been published, but those that have suggest it typically follows an exponential function. McKenna *et al.* ^38^ and Glendon ^39^ assessed individuals’ ability to perform cardiopulmonary resuscitation (CPR) after different intervals without practice, and both studies found that the data fitted an exponential decay curve. Recently, Candia *et al.* ^40^ examined data on papers, patents, songs, movies, and biographies and found that collective memory and attention decays biexponentially. In our study, we opted to assume that the loss of the knowledge and skills required to use wildfire to create a campfire followed an exponential decay curve, because CPR is more similar to the use of wildfire than are the activities that give rise to the types of data analyzed by Candia *et al.* ^40^. Importantly, there is no difference between the two curves at the tail of the decay, which is the crucial part for our model of cultural loss.

Ethnographically known foragers use a variety of social learning and transmission strategies ^41^. We deliberately did not consider the impact of different social learning mechanisms (e.g., vertical transmission *vs* horizontal transmission) on the process by which the hypothetical Neanderthal group may lose the ability to use the WCP. We focused on the total number of experts regardless of how they obtained their knowledge and skills. This made the model simpler. It also—like the return to the maximum number of experts each time the WCP was used—reduced the probability of the WCP being lost, which in turn reduced the probability that EMBERS would support Sandgathe and colleagues’ hypothesis ^14– 16^. This made our study conservative.

The last major assumption we made is that our hypothetical Neanderthal group could only utilize the WCP when they had direct access to wildfire and could not obtain embers in any other way if their campfire went out (e.g., by raiding neighboring groups as the Sirionó are known to have done ^9^). This assumption, which links the frequency of use of the WCP to the frequency of occurrence of wildfire (as opposed to, say, volcanic eruptions), is at the heart of Sandgathe and colleagues’ ^13– 16^ hypothesis. In the temperate zone of the Northern Hemisphere, wildfires are usually triggered by lightning but also depend on the availability of combustible material ^42^. Consequently, the occurrence of wildfire is spatiotemporally variable: it does not occur every year in a given location. In line with this, we modeled the timing of the use of the WCP with a series of normal distributions with different mean intervals and different variances around the mean interval.

## Results

We report results obtained with two versions of EMBERS, a numerical version and an analytical one. The numerical version was created to provide a clear picture of the different elements of the model. The analytical version was developed to allow faster exploration of the parameter space. An advantage of generating both versions of a model is that it enables cross-validation. We estimated the probability of a Neanderthal group losing the expertise necessary to use the WCP in a 1000-year length (L) in the face of fluctuations in (i) the mean interval between uses of the WCP ( *θ*); (ii) variability in the intervals ( *η* *ν*); (iii) the maximum number of experts in the group ( *i.e.*, the maximum number of individuals with the knowledge and skills necessary to use the WCP; *η ^max^*); and (iv) the decay on the number of experts in the group ( *τ*). The definitions, characteristics, and ranges of values of the parameters used in the simulations are summarized in Table 1 and discussed in detail below. When creating the two versions of EMBERS, we assumed that the parameters were independent of each other. This assumption is probably unrealistic but there are currently no data or theory that shed light on possible dependencies among the parameters. As such, we opted for the simplest assumption, which is independence among all parameters. We analyzed every combination of variables ( *θ, ν, η ^max^*, *τ*) for the values in the ranges outlined in the Materials and Methods section. For each combination of values, we computed the probability of retention, *P _r_*.

**Table 1.** Main parameters of the EMBERS model.

Parameter	Symbol	Definition	Units	Type	Values
*Time Length*	*L*	The maximum length of time modeled by EMBERS. Note that not all runs of the model get to *L* in the numerical version. Sometimes the ability to use the WCP is lost before *L* is reached. In such cases, the run ends when the ability to use the WCP is lost.	Years	Fixed	1000
*Temporal Sequences*	*K*	The number of sequences that are used for each combination of parameters in the numerical version of EMBERS. The number has to be high enough to obtain a probability distribution.	-	Fixed	111
*Use Interval*	*θ*	The mean of the normal distribution that is used to determine the intervals between uses of the WCP for a single temporal sequence and for the temporal sequences that comprise a simulation.	Years	Variable	1–20
*Variability*	*v*	A dimensionless scaling factor used to represent the uncertainty associated with the intervals between uses of the WCP.	-	Variable	0–2
*Maximum Number of Experts*	*η ^max^*	The maximum possible number of experts for a given temporal sequence. For the present study, an expert is an individual who has the knowledge and skills to use wildfire to create a campfire ( *i.e.*, someone capable of using the WCP). Each temporal sequence starts with the number of experts at *η ^max^*, and each time the WCP is used the number of experts returns to *η ^max^*.	Individuals	Variable	3–60
*Forgetting Time*	*τ*	The interval it takes for the number of experts to be reduced to about a third of *η ^max^* ( 1e≈0.36 ).	Years	Variable	1–16

*P _r_* is computed differently in the numerical and analytical versions of EMBERS but the calculations depend on the same set of assumptions and parameters, such that the two versions of *P _r_* provide comparable estimates of the probability of retaining the WCP in *L* for a given combination of variable values.

**Figure 1.**
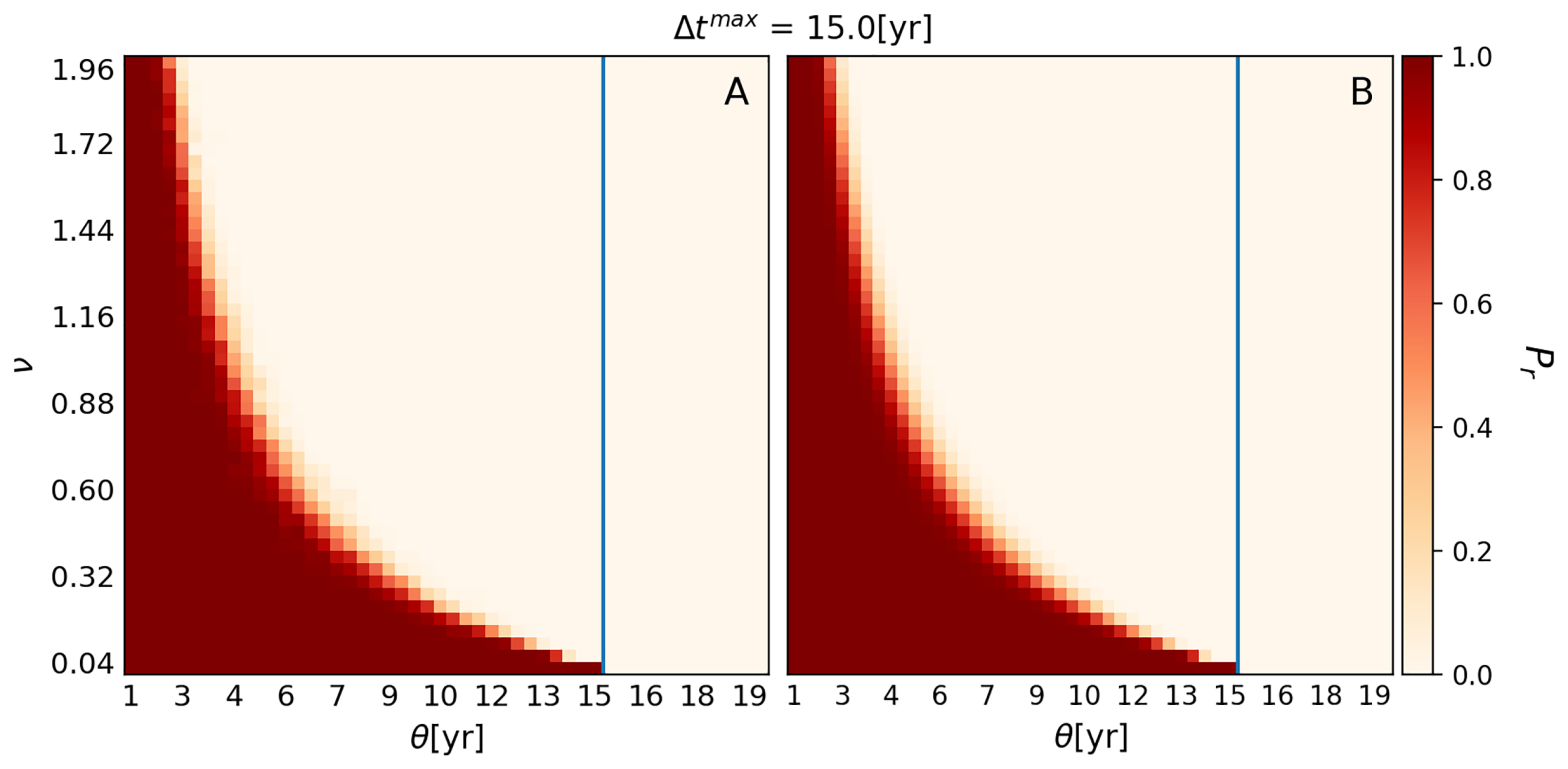
Plots showing *Probability of Retention* ( *P _r_*). *P _r_* for different combinations of *Use Interval* ( *θ*) and *Variability* ( *ν*) when *Forgetting Time* ( *τ*) and *Maximum Number of Experts* ( *η* ^max^) are fixed at 4 and 43, respectively. Plot **A** was generated with the numerical version of EMBERS, while Plot **B** was produced with the analytical version. Each square of the grid is color-coded to represent the probability that the WCP will be retained by the hypothetical Neanderthal group at the end of 1000 years. Dark red represents a high *P _r_*, while cream indicates a complete loss before 1000 years was reached. The pixels shaded in lighter red and orange represent combinations of variable values that result in intermediate *P _r_*. The lighter red-to-orange zone marks the transition between retention and loss of the WCP. The blue vertical line represents Δ *t **^max^***, which is 15 years for the chosen set of variable values.

Figure 1 shows *P _r_* for different values of *θ* and *ν*, when *η ^max^* and *τ* are fixed at 43 and 4, respectively. The first result to note is that all the squares to the right of the blue line marking Δ *t ^max^* show a complete loss of the WCP before *L* = 1000. This is expected because Δ *t ^max^* is the time it takes for the number of experts to drop to 0 and therefore is the upper limit for retention of the ability to use the WCP in the absence of the occurrence of a wildfire. It is simply not possible for the probability of retention ( *P _r_*) to be greater than 0 if *Use Interval* ( *θ*) is longer than Δ *t ^max^*.

Next, even allowing for the fact that the loss of the ability to use the WCP to the right of the blue line is expected, it is evident that there are many combinations of *θ* and *ν* that result in a complete loss of the ability to use the WCP during the 1000-year period modeled by EMBERS. If we focus on the combinations to the left of the blue line, approximately two-thirds of them result in the loss of the ability to use the WCP before *L* = 1000, while only one-third result in the retention of the ability to use the WCP up to *L* = 1000. Thus, Figure 1 indicates that our *in silico* Neanderthal group could lose the ability to use wildfire to make campfires even when wildfires were still occurring in its territory. Notably, Figure 1 suggests that the loss of the ability to use wildfire to make campfires was not just possible but in fact more likely than the retention of the ability.

Turning to the individual impact of the two variables, it is evident that *Use Interval* ( *θ*) has no effect on *P _r_* providing *Use Interval* is less than Δ *t ^max^*. This can be seen if we move from left to right along the bottom rows of Plots A and B, where *ν* is between 0 and 0.04. All the squares show a high probability of retention of the WCP for 1000 years, until the line showing the location of Δ *t ^max^* is reached. Beyond the line, all the squares show a failure to retain the WCP to 1000 years. The implication of this is that the only value of *θ* that is relevant for estimating *P _r_* is the one that corresponds to Δ *t ^max^*. Another way of thinking about this is that it is Δ *t ^max^* that dictates the boundary between the retention and loss of the WCP, rather than *θ*.

Unlike *θ*, *Variability* ( *ν*) has a substantial impact on *P _r_*. Moving upwards on the y-axis of both panels, we can see that increasing *ν* from 0 to 0.32 results in the boundary between retention and loss of the WCP changing from 15 years to about nine years. Then, increasing *ν* from 0.32 to 0.88 changes the boundary to approximately five years. Further increasing *ν* from 0.88 to 2, changes the boundary between retention and loss of the WCP to around three years. Thus, *ν* has a non-linear impact on *P _r_*, and the impact is such that increases in *v* have a much larger effect on *P _r_* when *v* is small than when *v* is large.

We will now examine the way *P _r_* is affected by the other two key variables, *τ* and *η ^max^*. Because *τ* and *η ^max^* affect *P _r_* via Δ *t ^max^* ( Equation 3 in the Materials and Methods section), we will begin by examining the influence of *τ* and *η ^max^* on Δ *t ^max^* using results from the analytical version of EMBERS.

**Figure 2.**
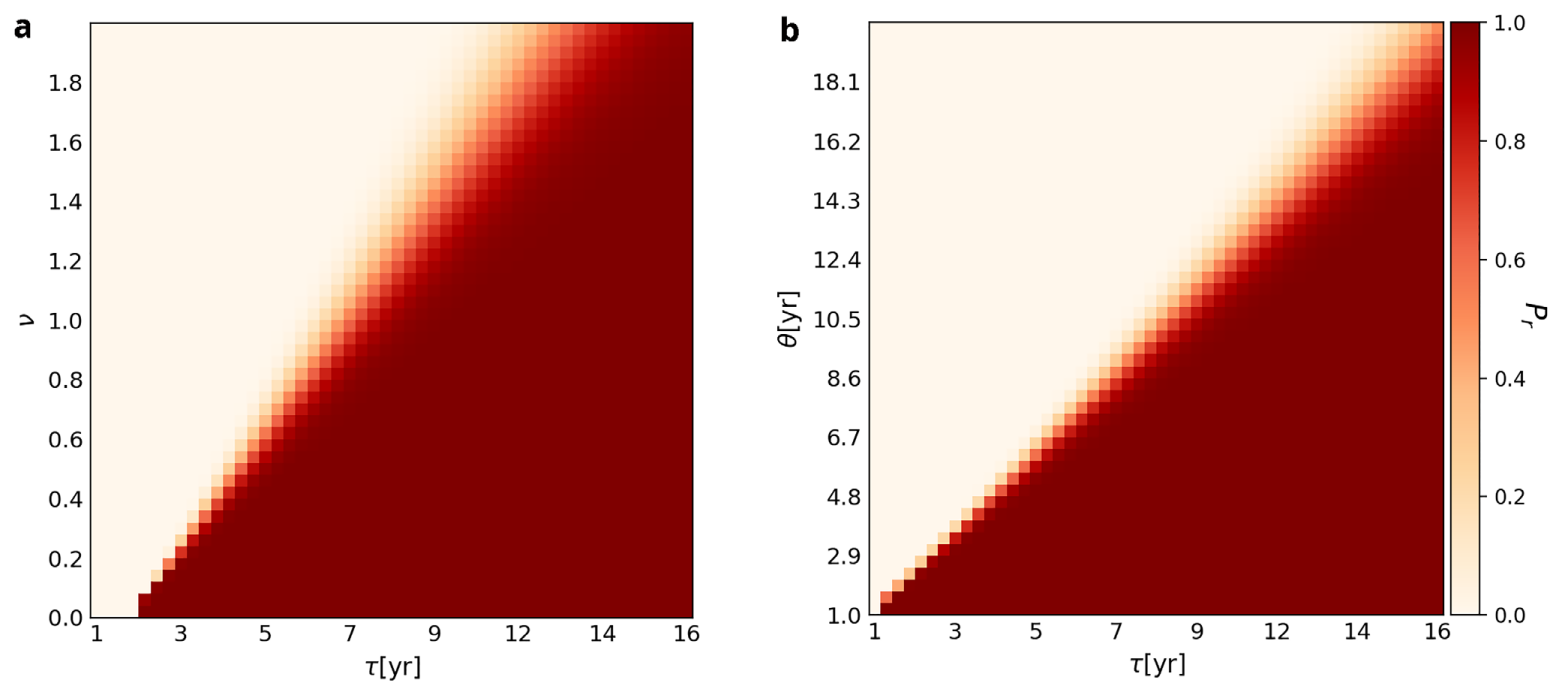
Probabilities of retention ( *P _r_*) associated with different combinations of *Forgetting Time* ( *τ*), *Variability* ( *v*), and *Use Interval* ( *θ*). *η ^max^* is set at 8. *θ* is fixed at 4 in Plot **A**, while *v* is fixed at 0.3 in Plot **B**. Each square of the grid is color-coded to represent the likelihood of retention of the WCP at the end of 1000 years. The color scheme is the same as the one used in Figure 1.

The nature of the relationship between Δ *t ^max^* and *τ* is discernible when *P _r_* is plotted in relation to different values of *v* and *θ*. The same holds for the nature of the relationship between *η ^max^* and Δ *t ^max^*. Figure 2 plots *P _r_* in relation to a sample of different values of *τ*, *v* and *θ*. In Plot A, the transition zone between retention and loss is linear. Thus, when *Variability* is low ( *e.g.*, *v =* 0.1), the ability to use the WCP can be retained in the face of a *Forgetting Time* as short as two years ( *τ <=* 2), whereas when *Variability* is high ( *e.g.*, *v =* 1.5), *Forgetting Time* has to be ten years or more ( *τ* >= 10yr) for the group to retain the ability to use the WCP for 1000 years. Interestingly, the transition zone widens as *v* increases. This is due to stochastic processes playing an increasingly important role as *Variability* ( *v*) and *Forgetting Time* (τ) increase. In other words, the wider the transition zone, the more chance plays a role in the retention of the ability to use the WCP in the 1000-year time.

Panel B shows the same pattern. The transition zone between retention and loss is not only linear but also widens as *θ* increases. The implication of the former is that the greater the value of *Use Interval* ( *θ*), the longer the *Forgetting Time* ( *τ*) has to be for the hypothetical Neanderthal group to retain the ability to use the WCP. For example, when *θ* = 4, retention happens for values of *τ* as low as three, whereas when *θ =* 14, retention only happens for values of *τ* equal to or greater than 12 years. Similar to Panel A, the widening of the transition zone indicates that chance plays a greater role in the retention *vs* loss of the ability to use the WCP as *θ* and *Forgetting Time* increase.

**Figure 3.**
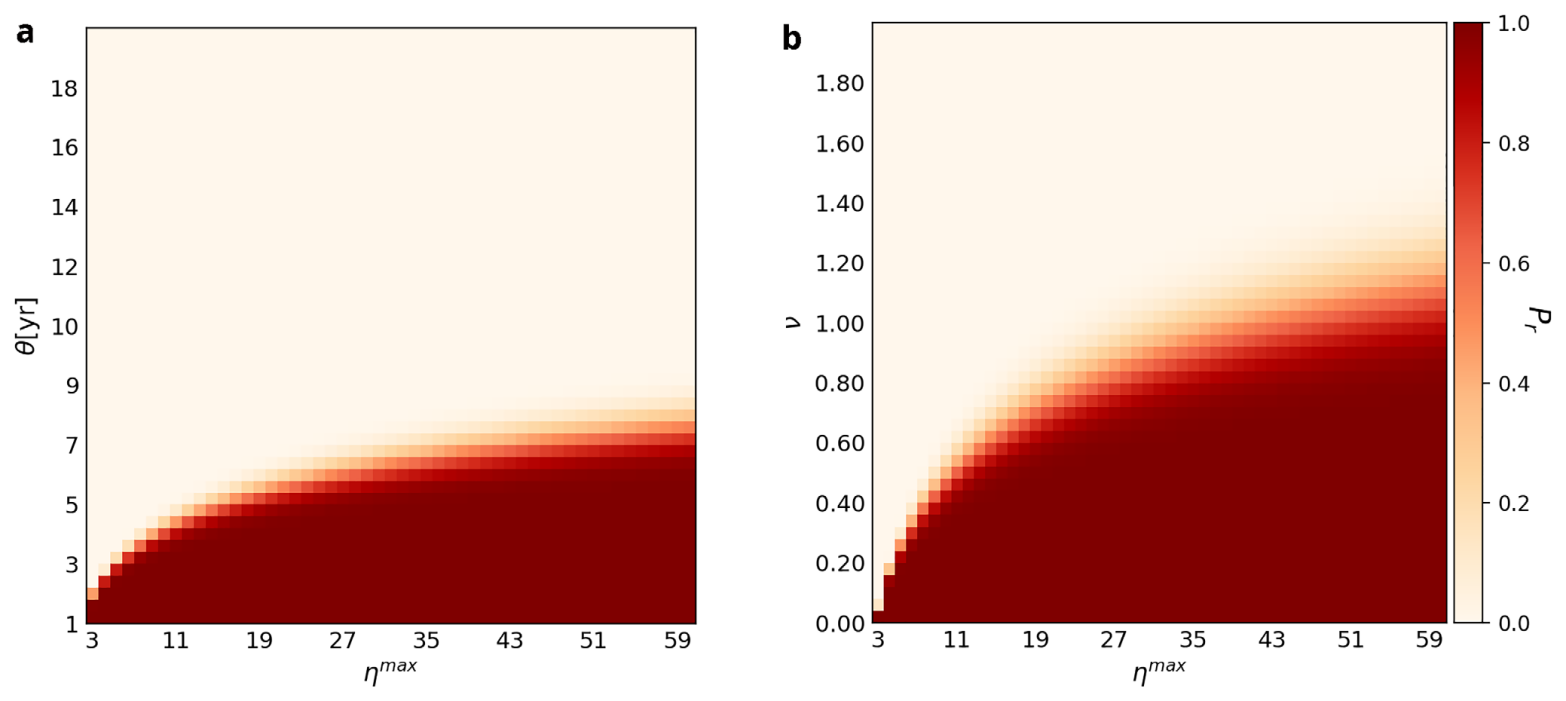
Retention probabilities for different combinations of *η ^max^* and *ν* and *η ^max^* and *θ*. *τ* is fixed at four in both panels, *θ* is set at 4 in Plot **A**, while *ν* is fixed at 0.3 in Plot **B**. Each square of the grid is color-coded to represent the likelihood of retention of the WCP at *L* = 1000. The color scheme is the same as in Figure 1 and Figure 2.

Figure 3 plots *P _r_* in relation to a sample of different values of *η ^max^*, *v*, and *θ*. In Panel A, the transition zone between retention and loss is a positive logarithmic curve and widens as *η ^max^* increases. The former means if the variability ( *v*) doubles, the number of experts has to quadruple in order to retain the ability to use the WCP for 1000 years. This effect can be appreciated by locating the values of *η ^max^* required to retain the ability to use the WCP in the face of values of *v* of 0.6 and 1.2. When *v* = 0.6, it is probably sufficient for the hypothetical Neanderthal group to have just 14 experts. However, when *v* is 1.2, the group must have 60 experts to retain the ability to use the WCP for 1000 years.

Plot B of Figure 3 shows that the relationship between *η ^max^* and *θ* is similar. The transition zone between retention and loss is not only a positive logarithmic curve but also widens as *θ* increases. The implication of the former is that if *Use Interval* doubles, then the maximum number of experts must quadruple in order for the ability to use the WCP to be retained until *L* = 1000. For example, if *θ* = 4, then *η ^max^* has to be ≥9 to retain the ability to use the WCP until *L* = 1000, whereas if *θ* = 6, then *η ^max^* has to be ≥30.

**Figure 4.**
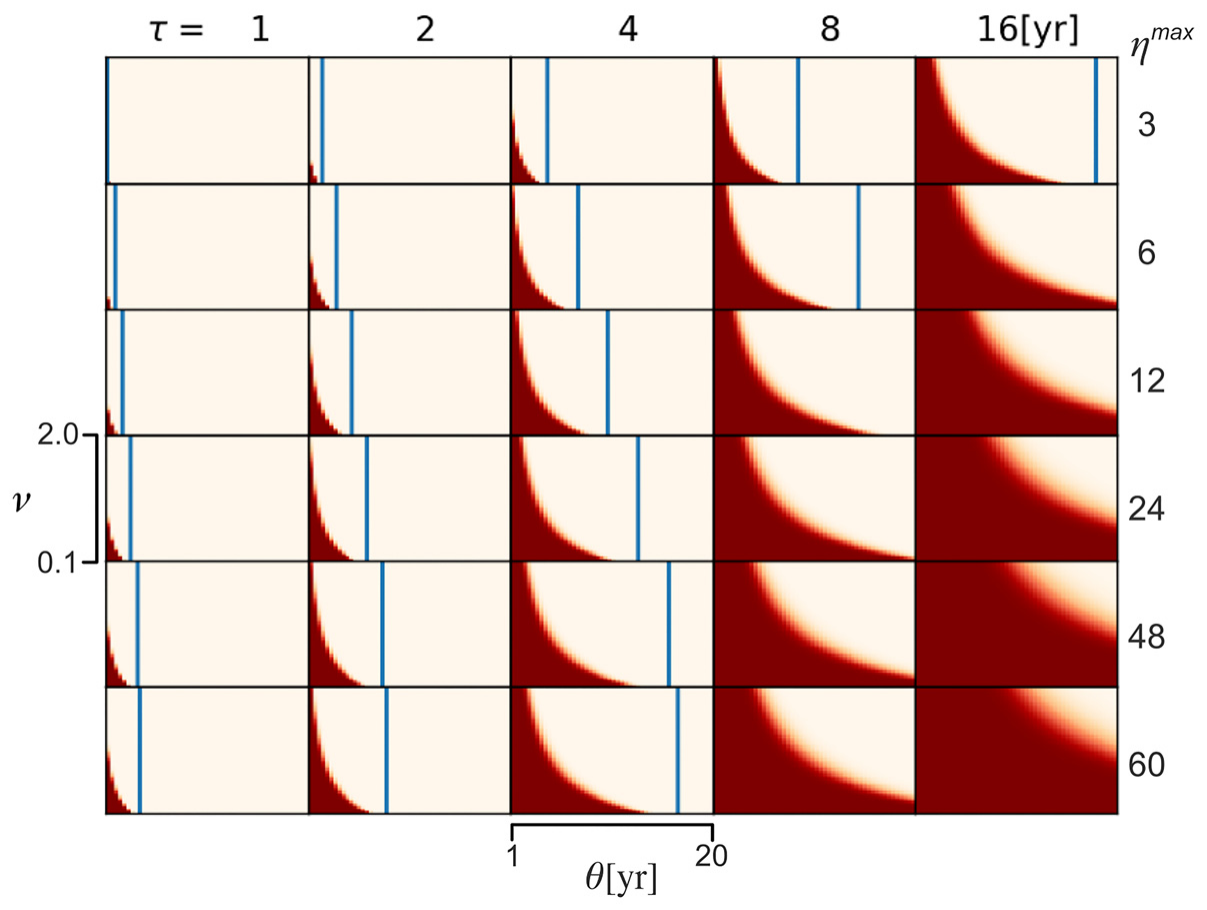
Multiplot showing the *Probability of Retention* ( *P _r_*). ***P _r_*** represented for different combinations of *Use Interval* ( *θ*), *Variability* ( *v*), *Forgetting Time* ( *τ*), and *Maximum Number of Experts* ( *η* ^*max*^). Each sub-plot shows *P _r_* for combinations of *θ* and *v* when *τ* and *η* ^*max*^ were varied. The brackets to the left of the fourth row and the bottom of the third column show the ranges of *v* and *θ,* respectively. For consistency, the ranges of values of τ and *η ^max^* are the same as those seen in Figure 1. The color-coding scheme is the same as in Figure 1, Figure 2, and Figure 3.

Figure 4 is a grid of plots that show the retention probability associated with a sample of different combinations of values of *θ*, *v*, *τ*, and *η ^max^*. The ranges for *θ* and *v* match those seen in Figure 1 and are used in all the plots in the grid. To make the Fig. less cluttered, only the minimum and maximum values of the ranges are shown. Each plot relates to a different combination of values of *τ* and *η* ^max^. The ranges of values of *τ* and *η ^max^* are τ = 1, 2, 4, 8, 16, and *η ^max^* = 3, 6, 12, 24, 48, 60. The color coding scheme for the squares within the plots is the same as in Figure 1, Figure 3, and Figure 4. Once again, the blue lines represent the Δ *t ^max^* for the modeled combination of variable values. Where a plot lacks a blue line, it means that the Δ *t ^max^* is larger than the relevant maximum value of *θ*.

As with Figure 1, it is clear from Figure 4 that there are many combinations of *τ* and *η ^max^* that result in a loss of the ability to use the WCP during the 1000-year time span. Again, this is the case even when we consider the fact that the loss of the ability to use the WCP to the right of the blue line is expected. In 25 of the 30 panels, the number of combinations of values of *τ* and *η ^max^* that result in the loss of the ability to use the WCP before *L* = 1000 is greater than the number of combinations of values of *τ* and *η ^max^* that result in the retention of the ability to use the WCP up to *L* = 1000. Thus, like Figure 1, Figure 4 does not merely suggest it is possible that the Neanderthal group could lose its ability to use wildfire to create campfires even though wildfires are still occurring on occasion. It suggests it is more likely that the group would lose the ability than that they would retain the ability.

The impact of *Forgetting Time* ( *τ*) on the probability of retention ( *P _r_*) can clearly be seen in Figure 4, as can the impact of *Maximum Number of Experts* ( *η ^max^*) and the combined effect of the two variables. Focusing on the fourth row of panels from the top (the one corresponding to *η ^max^* = 24), we can see that the larger the value of *τ* ( *i.e.*, the longer *Forgetting Time*), the greater the probability that the hypothetical Neanderthal group will retain the ability to use the WCP for 1000 years. Similarly, if we focus on the fourth column of panels from the left (the one corresponding to *τ* = 8), we can see that the probability of the group retaining the ability to use the WCP until *L* = 1000 increases as *η ^max^* increases. However, the effect of each of these variables is modified by the other variables. For example, when *Forgetting Time* is set at the shortest possible time ( *τ* = 1) and *Maximum Number of Experts* is at fixed at the lowest possible value ( *η ^max^* = 3), the hypothetical Neanderthal group never retains the ability to use the WCP for the 1000-year time. In contrast, when *Forgetting Time* is set at the longest possible time ( *τ* = 16) and *Maximum Number of Experts* is fixed at the maximum possible ( *η ^max^* = 60), the group nearly always retains the ability to use the WCP for 1000 years. Regarding the relative importance of *τ* and *η ^max^*, the impact of varying *τ* is greater than the impact of varying *η ^max^*. This can be appreciated by comparing the bottom row with the rightmost column. The change in the size of the dark red area, which denotes retention of the ability to use the WCP, is much greater as one moves from *τ* = 1 to *τ* = 16 in the bottom row than it is as one moves from *η ^max^* = 3 to *η ^max^* = 60 in the rightmost column. Thus, while both *Forgetting Time* and *Maximum Number of Experts* impact the probability of retention, *Forgetting Time* is the more influential of the two variables.

## Discussion

The study reported here was motivated by a debate about a counterintuitive pattern documented at multiple Neanderthal-linked archaeological sites in Europe—a decrease in evidence for fire use in layers deposited in colder conditions. Sandgathe and colleagues ^13– 16^ have proposed that this pattern indicates that European Neanderthals were unable to create fire and instead relied on wildfire to start their campfires. This hypothesis explains the decline in evidence of the use of fire in colder conditions, according to Sandgathe and colleagues ^13– 16^, because lightning strikes are more common in temperate conditions than in cold, dry ones and therefore so are wildfires. Critics of Sandgathe and colleagues’ ^13– 16^ hypothesis have argued that it is flawed because lightning strikes would not have stopped entirely as climatic conditions deteriorated, they just would have become less frequent and more irregular ^17^. It is not plausible, the critics contend, that European Neanderthals would have forgotten how to use wildfire to create campfires if they still encountered wildfire on occasion and therefore other explanations must be considered. The goal of the present study was to shed light on this debate about the plausibility of Sandgathe and colleagues’ hypothesis. To do so, we developed a model that we call EMBERS.

EMBERS estimates the probability of a hypothetical group of Western European Neanderthals losing the knowledge and skills required to use wildfire to create a campfire, *i.e.*, the ability to use the wildfire-use cultural package (WCP). Specifically, it estimates the probability of losing the WCP in a 1000-year period in the face of variation in (i) the maximum number of individuals able to use the WCP ( *Maximum Number of Experts*); (ii) the rapidity of decay of the group’s ability to use the WCP ( *Forgetting Time*); (iii) the time between uses of the WCP ( *Use Interval*); and (iv) the uncertainty associated with the preceding variable ( *Variability*). In the study, we grounded EMBERS in the empirical world by utilising values for *Maximum Number of Experts* that were drawn from studies that have estimated Neanderthal group size, and values for *Forgetting Time* that were taken from studies dealing with the loss of procedural-motor skills. In a similar vein, we utilised values for *Use Interval* and *Variability* that reflect the known temporal dynamics of wildfires in the northern hemisphere’s temperate zone. In order to cross-validate the results, and for computational efficiency, we created two versions of EMBERS, a numerical version and an analytical version.

The results yielded by the two versions of EMBERS were consistent: The loss of the ability to use wildfire to create campfires is a more likely outcome than retention of the ability when the variable parameters were assigned values that approximate the conditions assumed by Sandgathe and colleagues ^13– 16^, i.e., when the frequency and regularity of wildfires declines. In fact, the results indicate that the loss of the ability to use wildfire to create campfires is a more likely outcome than its retention for a large majority of the potential combinations of values of the four variables. Thus, our study supports the plausibility of Sandgathe and colleagues’ ^13– 16^ hypothesis. Continuing to encounter wildfire would not necessarily have ensured that the Neanderthal groups were able to maintain the ability to use wildfire to create campfires. The speed of decay of procedural-motor skills is such that, for groups that depended on access to wildfire to start their campfires, it would only have taken a relatively small decline in the frequency and regularity of wildfires for the groups to have lost the relevant cultural knowledge.

The foregoing results are, of course, dependent on EMBER’s assumptions. However, there is no reason to believe that these made the results unreliable. We made seven main assumptions when creating EMBERS: ( *i*) the primary cause of wildfire is lightning; ( *ii*) campfires last for only a short period, *i.e.*, a few days or weeks; ( *iii*) the process of using wildfire to create and curate a campfire is sufficiently complex that it must be the product of social learning and cultural transmission; ( *iv*) the WCP involves procedural-motor skills and therefore undergoes exponential decay like the procedural-motor skills whose retention has been investigated in present-day *H. sapiens*; ( *v*) the hypothetical Neanderthal group permanently loses the WCP if the number of experts drops below 1; ( *vi*) the number of experts recovers instantly to the selected value for *Maximum Number of Experts*, after a use of the WCP; and ( *vii*) the values of the parameters do not change within a given simulation. Assumptions *i, ii, iii*, and *iv* are grounded in empirical evidence. The remaining three assumptions are harder to defend, but it is unlikely that they biased the results in favor of loss of the ability to use the WCP. Assumption *v* increases the probability of the WCP being lost, but assumptions *vi* and *vii* promote retention of the WCP. So, if assumptions *v*, *vi*, and *vii* biased the results in a particular direction, they probably did so in favor of retention of the ability to use the WCP.

The results are also dependent on the values selected for the variable parameters. The values for the four variables were chosen in light of findings of empirical studies, and where it was necessary to make a call about the values at either end of the range for a variable, we selected values likely to result in a higher probability of retention. Thus, if anything, the values we selected for the variables biased the results in favor of retention of the ability to use the WCP.

It appears, then, that we can invest reasonable confidence in the finding that it is more likely that the ability to use the WCP would be lost than retained in conditions akin to those experienced by European Neanderthal groups during cold, dry periods of the Pleistocene. This means that our study suggests Sandgathe and colleagues’ ^13– 16^ explanation for the pattern of fire evidence at Pech de l’Azé IV, Roc de Marsal, and several other Neanderthal sites in Europe is plausible. That is, it suggests the decline in fire evidence at the sites in question after climatic conditions worsened *could* be due to the Neanderthals who occupied them being unable to create and curate fire and having to rely on wildfire to create campfires. Contrary to what critics have argued ^17^, continuing to encounter wildfire would not necessarily have ensured that the Neanderthal groups were able to maintain the ability to use wildfire to create campfires. The speed of decay of procedural-motor skills is such that it would only have taken a small decline in the frequency and regularity of wildfires for the groups to have lost the relevant cultural knowledge.

Although the EMBERS model was developed to evaluate a hypothesis regarding the pyrotechnological abilities of European Neanderthals, none of the assumptions it makes is specific to European Neanderthals, or even Neanderthals in general. The assumptions hold for any group of hominins using wildfire to create campfires in the temperate zone of the Northern Hemisphere, including *H. sapiens*. Indeed, given that lightning is the primary cause of wildfire worldwide, and that the frequency and predictability of lightning strikes vary through time in all regions of the world, the assumptions hold for any group of hominins reliant on wildfire to create campfires. One implication of this is that we should be prepared for the possibility that, prior to the development of the ability to make fire from scratch and means to keep that knowledge from vanishing, the use of fire was often a temporary phenomenon. The results of the present study suggest that, prior to the development of the ability to make fire from scratch, it may have been common for hominin groups to gradually develop the ability to use wildfire to create campfires via cumulative cultural evolution and then rapidly lose the ability due to a change in the local wildfire regime. This in turn implies that wildfire-dependent hominin groups would have found it difficult to persist after migrating into temperate environments unless they had other means of coping with cold temperatures such a high basal metabolic rate or clothing.

The present study also has implications for the ongoing effort to develop an adequate theory of cultural evolution ^43^. Researchers interested in cultural evolution have discussed cultural loss, but they have done so primarily in the context of trying to elucidate the relationship between cultural complexity and demography (e.g., 44– 53). Little attention has been paid to the importance of cultural loss relative to cumulative cultural evolution, or to the specific mechanics of cultural loss. The results yielded by EMBERS indicate that this is unfortunate. That the loss of the WCP was more common than its retention suggests that cultural loss has the potential to be a highly influential process, and that we need to change how we think about cultural evolution. We should view humans and other cultural species as having to constantly contend with the decay of their knowledge and skills and treat the maintenance of existing cultural traits as at least as great a challenge as the invention and transmission of new ones. Regarding the mechanics of cultural loss, EMBERS shows that memory decay is critically important. The rate of memory decay varies by the type of cultural trait (e.g., procedural skills tend to be forgotten faster than perceptual skills ^54^ but in the absence of ways of countering memory decay, all cultural traits will degrade and eventually be lost, resulting in a reduction in cultural richness and/or complexity. This implies that memory decay should be recognized as a key cultural evolutionary process alongside copying error, guided variation, and the various types of cultural transmission that have been recognized. An obvious corollary of this is that the main ways of countering memory decay should also be treated as important phenomena by cultural evolutionary theorists. So far, memory decay and ways of countering it have received little attention in the cultural evolutionary literature. We have only been able to identify three recent relevant publications—Wakano and Kadowaki ^55^, which included a rate of loss of skill; Ammar *et al.* ^56^, which focused on the related process of forgetting ( *i.e.*, deliberate, active erasure of knowledge); and Morin ^57^, which did not explicitly discuss memory decay but did discuss techniques that human groups employ to retain traditions, including repetition and redundancy. Based on the results of the present study, there is, we suggest, good reason for researchers interested in cultural evolution to increase the number of studies dealing with cultural loss and ways of retaining knowledge and skills.

As we indicated in the Introduction, implausibility due to wildfire being less frequent rather than non-existent in cold conditions is not the only grounds on which Sandgathe and colleagues’ ^13– 16^ hypothesis has been criticized. Two other criticisms can be identified in the literature. One is that there are better explanations for the empirical finding that Sandgathe and colleagues ^13– 16^ developed their hypothesis to explain—i.e., the dramatic decrease in evidence for fire use in layers deposited in colder conditions at multiple Neanderthal-linked sites in Europe ^17, 58^. (The other criticism is that there is archaeological evidence that demonstrates Neanderthals were able to create fire at will ^12, 59^. While these criticisms are not directly relevant to the goal our study, which was to assess the plausibility of Sandgathe and colleagues’ ^13– 16^ hypothesis, we will briefly comment on them.

Alternative explanations for the decline in fire evidence highlighted by Sandgathe and colleagues ^13– 16^ have been put forward by Henry ^58^ and Sorensen ^17^. Henry ^58^ focused on the economics of fire creation and use and proposed that the Neanderthal groups in question stopped making fire because the calories they expended collecting the resources needed to create and maintain a fire had started to exceed the extra calories obtained from cooking food compared to eating it raw. Although we believe Henry was right to draw attention to the fact that the costs of making fire need to be considered alongside its benefits, we do not think her explanation for the pattern of fire evidence is better than the one put forward by Sandgathe and colleagues ^13– 16^. The reason for this is that Henry’s hypothesis assumes that the knowledge and skills required to create, maintain, and transport fire can linger unused for long periods of time and still be available when conditions change and the benefits of using fire start to exceed the costs again. This assumption is problematic. The relevant knowledge and skills can be expected to be subject to decay just like the knowledge and skills involved in CPR. Hence, a Neanderthal group that stopped making fire for an extended period of time because it had become uneconomic would likely lose the ability to create fire at will, just like the hypothetical Neanderthal group on which EMBERS focused lost the ability to use the WCP in many of the temporal sequences. Thus, the economic view of fire use promoted by Henry is incomplete. It is important to consider the costs and benefits of using fire, but that is not enough. The cultural processes involved, especially cultural loss, have to be considered too.

Sorensen ^17^ argued that the decline in fire evidence highlighted by Sandgathe and colleagues ^13– 16^ can be explained by the relevant Neanderthal groups adapting to colder, drier conditions by using smaller, short-lived fires for specific tasks. They would have done so, he argued, because woody fuel is less abundant in colder, drier conditions. According to Sorensen ^17^, the use of short-lived fires would have resulted in a significant decline in evidence for fire. This ‘ephemeral fire hypothesis’ was rejected by Sandgathe and colleagues ^15^ on the grounds that the artifact density at some of the relevant sites was so high that even small, short-lived, infrequent fires would have left burned artifacts. There are other problems with the ephemeral fire hypothesis. One is that it is inconsistent with the available ethnographic evidence, which, as we explained earlier, indicates that hunter-gatherers prefer to transport embers between camps rather than restarting fires from scratch ^9^. Another problem is that the ephemeral fire hypothesis, like Henry’s ^58^ economic hypothesis, presupposes that the knowledge and skills required to start a fire can be maintained by a group for any length of time. As the analyses reported here show, this is not a reasonable assumption. The relevant knowledge and skills can be expected to have decayed, and to have done so rapidly, if the group did not use them. Thus, even if the other problems with the ephemeral fire hypothesis are ignored, the ephemeral fire hypothesis is, like Henry’s ^58^ hypothesis, incomplete. Again, cultural loss and other cultural processes have to be considered.

The other criticism of Sandgathe and colleagues’ ^13– 16^ hypothesis that can be identified in the literature is that it cannot be correct because there is evidence that indicates Neanderthals were able to create fire at will. This criticism has appeared in two studies ^12, 59^ —Sorensen *et al.* (2018) and Brittingham *et al.* (2019). Sorensen *et al.* ^12^ claimed to have found microwear evidence that Mousterian bifaces (one of the key components of the Mousterian of Acheulean Tradition [MTA] as defined by François Bordes ^60^) were used to create fire. Sorenson *et al.* (2018) ^12^ argued that Neanderthals used MTA bifaces in conjunction with chunks of pyrite (FeS _2_) to produce fire. Specifically, they argued that Neanderthals struck pieces of pyrite against the flat/convex surfaces of MTA bifaces to produce sparks capable of setting tinder alight. They based this claim on a comparison between microwear they identified on a sample of MTA bifaces and microwear they generated in replicative experiments. This is an interesting hypothesis. However, Sorensen *et al.* (2018) ^12^ did not identify any evidence of pyrite on the bifaces they examined. Nor did they adequately investigate alternative causes of the damage they documented on the flat/convex surfaces of the bifaces in their sample. Equally problematically, Sorenson *et al.* (2018) ^12^ did not blind the experiments: the experimental microwear was created by a person who knew what they needed to produce to support the authors’ preferred explanation for the microwear on the archaeological artifacts, which means the results of the experiments cannot be trusted. Given these problems, Sorenson *et al.*’s (2018) ^12^ claim is unconvincing.

Brittingham *et al.* (2019) ^59^ approached the problem of trying to identify evidence that Neanderthals were able to create fire at will in a different way. They analyzed polycyclic aromatic hydrocarbons (PAHs) at the Armenian Middle Palaeolithic site of Lusakert Cave. PAHs are organic compounds that are produced when organic material is burned. Heavy PAHs are a major component of burned wood PAH emissions, while light PAHs are a major component of wildfire PAH emissions. Brittingham *et al.* (2019) ^59^ reported finding no association between the abundance of heavy PAHs and the abundance of light PAHs. Instead, they found that the abundance of heavy PAHs was correlated with the density of Middle Palaeolithic artifacts. They concluded from this that the Neanderthals who occupied the site must have been able to create fire from scratch and therefore were not dependent on wildfires to create their campfires. On the face of it, Brittingham *et al.*’s (2019) ^59^ results represent a substantial challenge to Sandgathe and colleagues’ ^13– 16^ hypothesis—and by extension this study. However, the data that Brittingham *et al.* (2019) ^59^ present in their Figure 1 clearly shows a positive correlation between the abundance of heavy PAHs and a proxy of environmental temperature, *δ*D _Wax_. This correlation indicates that heavy PHAs were more abundant in warmer conditions than in colder conditions. If heavy PHAs are indicative of campfires, as Brittingham *et al.* (2019) ^59^ contend, then their data are in line with Sandgathe and colleague’s ^13– 16^ hypothesis rather than inconsistent with it. Thus, Brittingham *et al.*’s (2019) ^59^ claim to have identified evidence that Neanderthals were able to create fire from scratch is also unconvincing.

In sum, then, the other two criticisms of Sandgathe and colleagues ^13– 16^ hypothesis are no more compelling than the claim that the hypothesis is implausible because Neanderthals would still have encountered wildfire when conditions became colder and drier.

With respect to future directions, in the next phase of our work we intend to extend EMBERS to explore a more complex scenario than the one examined in the present study. Earlier we noted that McCauley *et al.* (2018) ^9^ found that some of the ethnographically documented hunter-gatherer groups in their sample had lost the ability to make fire from scratch and therefore tried to obtain embers from neighbouring groups via trade or raiding. That the groups in question had lost the ability to create fire de novo cannot be easily explained by memory decay, because in principle it should have been possible for the groups to counter any loss of knowledge and skills simply by starting fires more frequently. A more likely explanation, we think, is that only a few group members had the ability to create fire from scratch and these individuals died as a result of, say, warfare or infectious disease. This raises the possibility that the present study overestimated the probability of retention of the WCP because we did not allow the relevant knowledge and skills to be lost by disappearance of experts as well as by memory decay. On the other hand, the observation that some of the groups who had forgotten how to make fire obtained embers from neighbouring groups raises the possibility that a wildfire-dependent hominin group could counter the effects of a change in the local wildfire regime by obtaining embers from another group. As we modeled an isolated Neanderthal group, EMBERS may have underestimated the probability of retention of the WCP. Unfortunately, it is not obvious how to think about the scale of the effect of including two processes leading to the loss of the WCP ( *i.e.*, memory decay and the disappearance of experts) versus the scale of the effect of including two sources of embers ( *i.e.*, wildfire and neighbouring groups). It requires another modelling study that extends EMBERS such that (i) the WCP is lost by both memory decay and the disappearance of experts, and (ii) the Neanderthal group is part of a network of groups and therefore may be able to obtain an ember from a neighbouring group, if one of them has a campfire in the relevant time frame.

## Materials and methods

### The numerical version of EMBERS

We created two versions of EMBERS, a numerical version and an analytical version. In this section, we outline the numerical version and justify our estimations of the parameter ranges based on the available literature and practical considerations.

### Main parameters and operations

To generate the *n* temporal sequences that comprise a numerical simulation, EMBERS performs three operations: It (i) determines the timing of uses of the WCP in each temporal sequence; (ii) establishes the amount by which the hypothetical Neanderthal group’s ability to use the WCP decays between uses; and (ii) decides whether the group will recover the ability to use the WCP or lose it permanently. (iv) Subsequently, the *n* temporal sequences are used to estimate the probability of the group losing the ability to utilize the WCP during each simulation. In this section, we will explain how each of these operations is carried out.

### Determining the timing of uses of the WCP

Each temporal sequence of a simulation comprises a series of intervals, which are measured in units of years. The first interval of each temporal sequence is the time between the start of the temporal sequence and the initial use of the WCP. The other intervals in a temporal sequence are the time between two uses of the WCP. Intervals are labelled as *i* and defined as $\text{Δ}t_{i}^{k}$ .

For each temporal sequence, $\text{Δ}t_{i}^{k}$ intervals are drawn from the positive values of a normal distribution generated with the following expression:

$$N(\theta ,{\left[\nu \cdot \theta \right]}^{2}),\;(1)$$

where *θ* is the mean in units of years and *v* is a parameter we call *Variability*, which is a dimensionless scaling factor used to represent the uncertainty associated with the intervals between uses of the WCP.

EMBERS generates 2 *L*/ *θ* values of, $\text{Δ}t_{i}^{k}$ *i.e.*, $i\in \left[1,\frac{2L}{\theta }\right]$ , for a given temporal sequence. For an unbiased normal distribution, there are, on average, *L*/ *θ* intervals. Thus, generating 2 *L*/ *θ* values of $\text{Δ}t_{i}^{k}$ ensures that the sum of the intervals will equal or exceed *L*. The consecutive draws of $\text{Δ}t_{i}^{k}$ for a temporal sequence are independent of each other.

Because the intervals that comprise a temporal sequence are drawn from a normal distribution, the intervals will vary in length even though the same *θ* and *v* are used to generate them. Most of the intervals will be close to the *θ* selected for the temporal sequence, but some will be substantially shorter, and others will be much longer ( Figure 6). The probability of drawing a $\text{Δ}t_{i}^{k}$ value that is very short or very long compared to *θ* is dependent on *v*. And the value of $\text{Δ}t_{i}^{k}$ is, in turn, dependent on the value of *ν* selected for the temporal sequence. The larger the value of *ν*, the greater the probability of drawing $\text{Δ}t_{i}^{k}$ that deviates greatly from *θ*.

**Figure 5.**
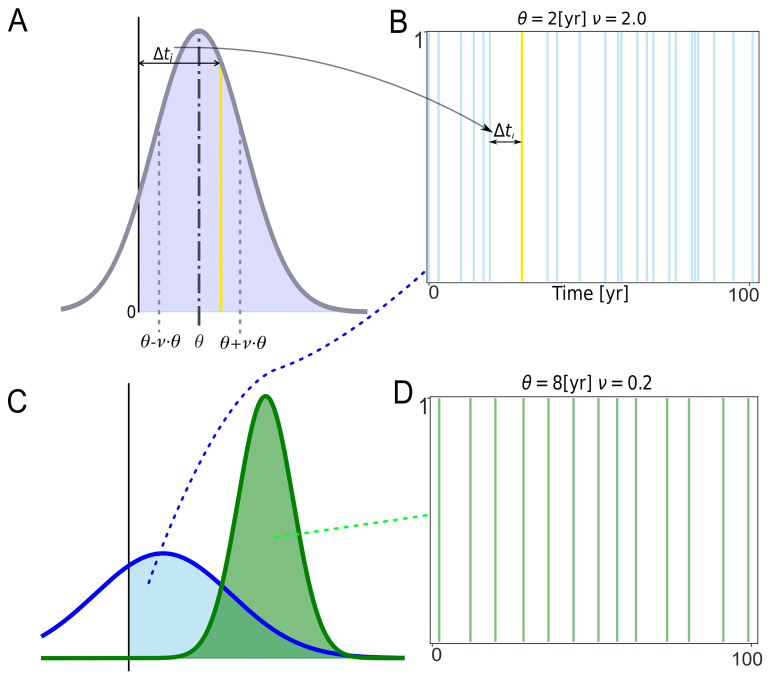
Diagram illustrating how intervals between uses of the WCP ( $\text{Δ}t_{i}^{k}$ ) are determined and different temporal sequences for two Normal Distributions. The intervals are drawn from the positive values of a normal distribution. The *θ* and *v* of the normal distribution are held constant for all the intervals in a temporal sequence and for all the temporal sequences in a simulation but are allowed to vary among simulations. Panel **A** illustrates the selection of a single value of *Δt _i_*. The normal distribution in **A** has a *θ* of 4 and a *v* of 1. The grey dotted lines correspond to the standard deviation of the normal distribution ( *θ* · *v*). The violet shaded area corresponds to the positive values of the normal distribution. The yellow line represents a focal use of the WCP (or any other complex practice), while the black line, marked with 0, represents the preceding use of the WCP. Panel **B** shows where the focal use of the WCP fits in a 100 year-run of the simulation. The blue vertical lines represent other uses of the WCP in that sequence. The intervals associated with these uses of the WCP were drawn from the blue normal distribution in Panel **C**. Panels **B** and **D** show the impact of different values of *θ* and *v*. The sequences in **B** and **D** were generated from the positive values of the normal distributions (green and blue shaded areas) shown on panel **C**. The green one has *θ* = 8 and *v* = 0.2, while the blue one has *θ* = 2 and *v* = 2. The positive values of the two distributions yield sequences of uses of the WCP that have different statistical properties. Most significantly for present purposes, the uses of the WCP in panel **D** are more regular than those in panel **B**.

While the values of *θ* and *v* are held constant for the temporal sequences included in a single simulation, the values of *θ* and *v* are varied between simulations. The effect that different values of *θ* and *v* have on the time between uses of the WCP (or other complex practices) is illustrated in Figure 5, which shows a segment of a sequence of uses generated with the aid of a normal distribution with *θ* = 8 and *v* = 0.2 and a segment of a sequence of uses based on a normal distribution with *θ* = 2 and *v* = 2.

**Figure 6.**
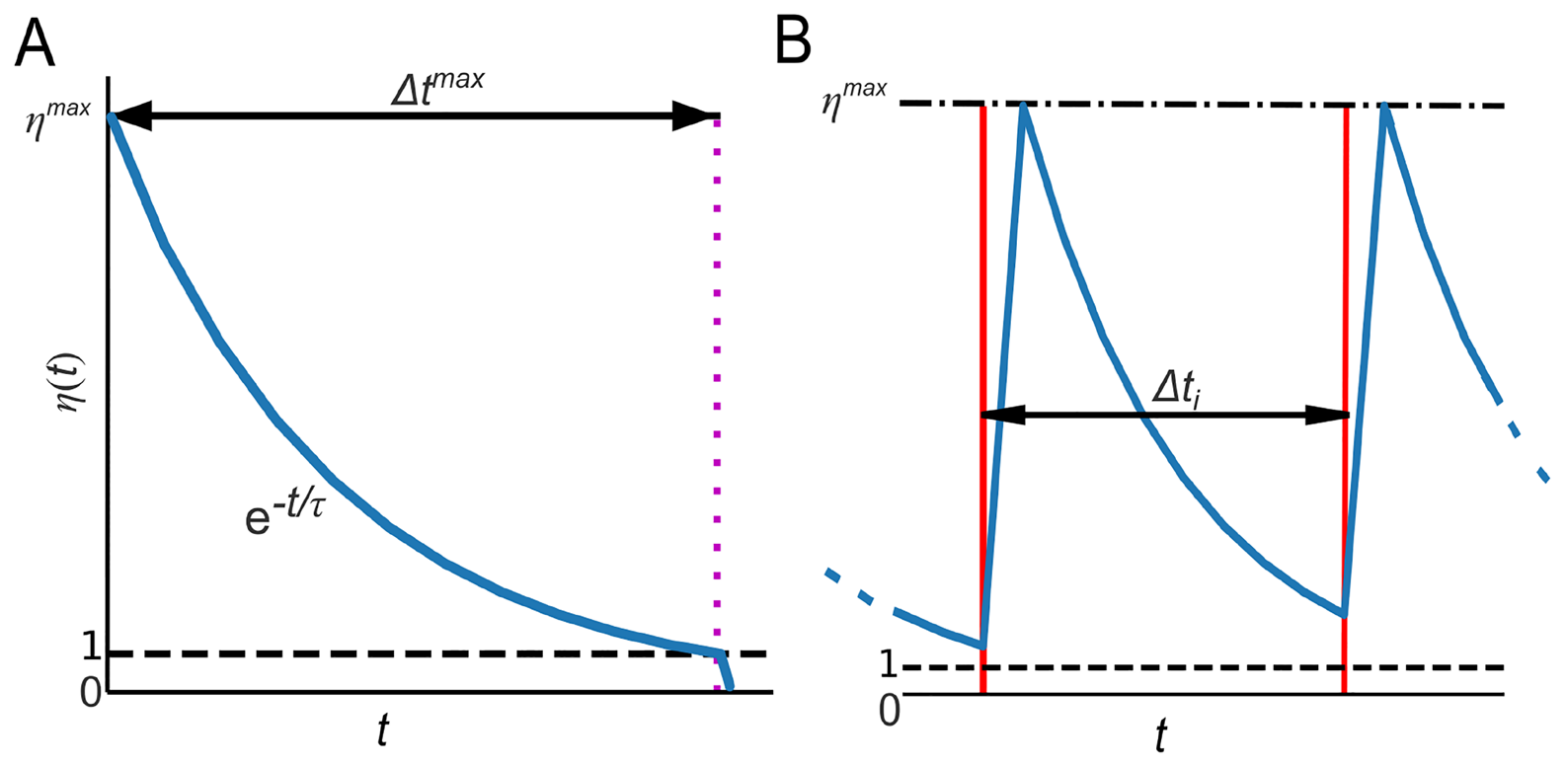
The decay and recovery of the hypothetical group’s ability to use the WCP. Panel **A**: the number of experts *η* over time (blue line) begins at *η ^max^* and then decays exponentially until a use of the WCP occurs or the number of experts drops below the specified minimum threshold (black dashed line). The time it takes for the number of experts to drop below the minimum threshold is denoted by Δ *t ^max^*. In the diagram, Δ *t ^max^* is marked by the magenta dotted line. Panel **B** represents a segment of a single run of a temporal sequence, the red lines are uses of the WCP (or any other complex practice). The black double-ended arrow shows the interval between the first use of the WCP and the second use ( *Δt _i_*) as seen in Figure 5. The black dashed and dotted line and the black dotted line represent the maximum possible number of experts ( *η ^max^*) and the minimum threshold for the number of experts, respectively. Moving from right, starting at *t* = 0, we see that the number of experts decreases exponentially. Then, after the first use of the WCP, the number of experts increases rapidly to *η ^max^*. Subsequently, the number of experts begins to decay again and does so until the second use of the WCP. At that point, the number of experts once again increases rapidly to *η ^max^*, before beginning to decay once more.

We created *K*=111 simulations using different combinations of values for *θ* and *v* to explore the impact of different values for *Use Interval* and different levels of the variability associated with *Use Interval*. We employed values of *θ* between 1 and 20[yr] and values of *ν* between 0.1 and 2.0.

We set the lower limit of *θ* at 1[yr] because our aim was to model the use of the WCP (and other complex practices) in the temperate zone following natural annual cycles. We set the upper limit of *θ* at 20 [yr] to represent the use of the WCP as a once-in-a-generation event. This was, we reasoned, the longest interval between uses consistent with a strong test of Sandgathe and colleagues’ ^13, 15, 16^ hypothesis.

The *v* values we selected were intended to represent a spectrum of wildfire regimes in the temperate zone of the northern hemisphere (or other spectrum of variable uses, like wale strandings, Pinetree blossoming or El Niño/La Niña events), which run from relatively predictable to highly erratic ^61, 62^. The lower limit of *v* corresponds to a variability of 10% of *θ*. This means that if, for example, *θ* is four years, the variability is 0.4 of a year. Such a level of variability results in just minor departures from the mean interval of four years. Specifically, it results in around 16% of the intervals between uses being ≥4 years. The upper limit of *ν* corresponds to a variability of twice of *θ*. This results in a much greater departure from the interval specified by *θ*. Returning to the previous example, if *θ* = 4 and *ν* = 2, then approximately 16% of the intervals between uses will be ≥12 years, so a relatively frequent event (on average) might experience long periods without use once every six times.

### Establishing the decay of experts in a

**Figure 7.**
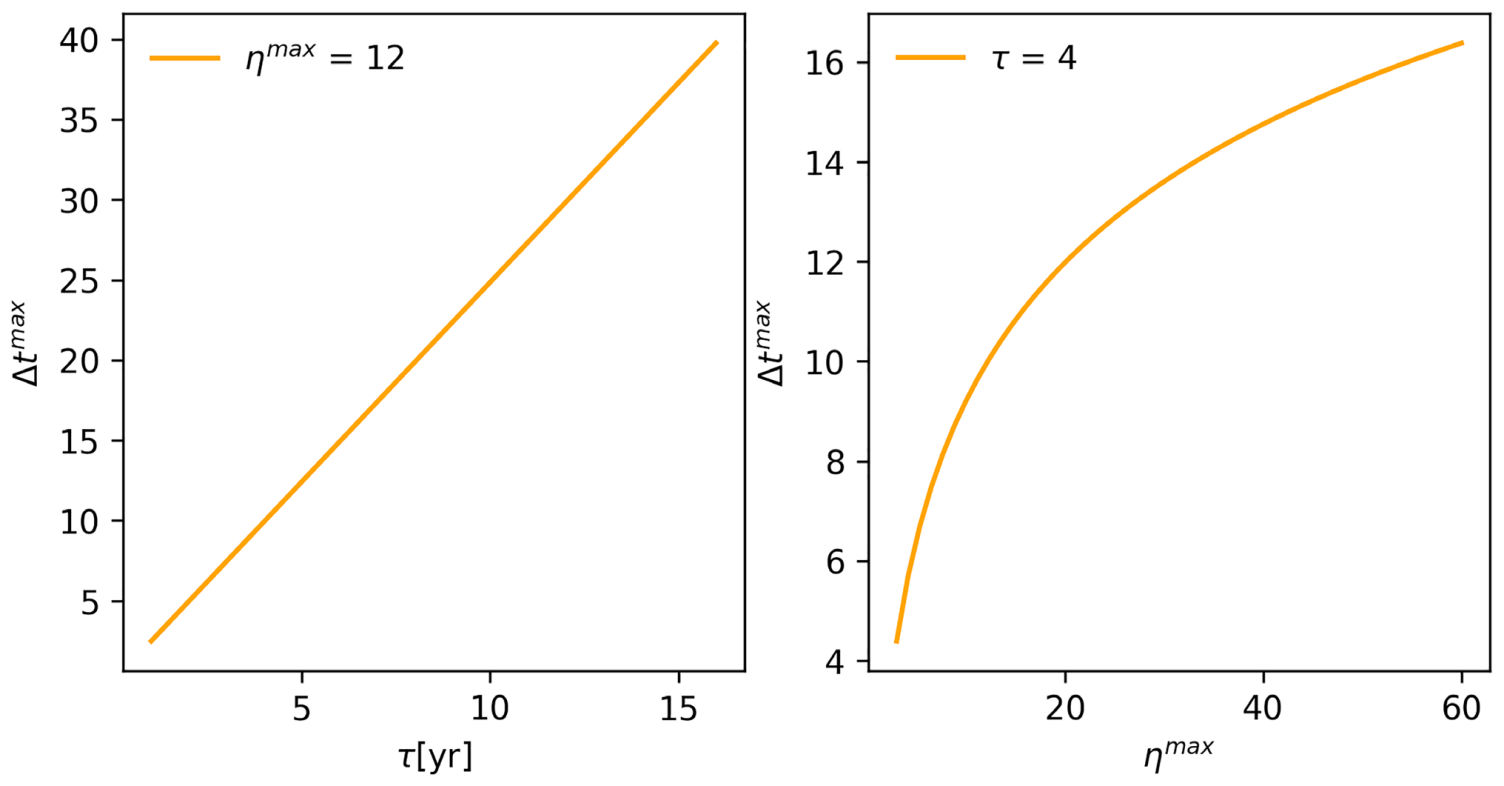
( **A**) Relationship between *Maximum Time* (Δ *t ^max^*) and *Forgetting Time* ( *τ*), and ( **B**) *Maximum Number of Experts* ( *η ^max^*). Plot **A** shows that Δ *t ^max^* is linearly dependent on *τ,* when *η ^max^* = 12. Plot **B** shows that Δ *t ^max^* is logarithmically dependent on *η ^max^*, when *τ* = 4. The relationships are of the same type ( *i.e.*, linear and logarithmic) when other values of *η ^max^* and *τ* are selected.

For each interval ( $\text{Δ}t_{i}^{k}$ ), the numerical version of EMBERS computes the number of group experts, denoted as *η*( $\text{Δ}t_{i}^{k}$ ). In line with the assumption that the number of experts decreases exponentially when not practicing, *η*( $\text{Δ}t_{i}^{k}$ ) is defined as:

$$\eta (\text{Δ}t_{i}^{k})=\eta^{max}exp(-\text{Δ}t_{i}^{k}/\tau ),\;(2)$$

where *η ^max^* is the maximum number of experts for a given temporal sequence, and *τ* is *Forgetting Time* ( Figure 7). To reiterate, an expert is someone with the knowledge and skills to use wildfire to create a campfire (or any other complex skill). *Forgetting Time* is the interval it takes for the number of experts to be reduced to about one-third of *η ^max^* ( 1e≈0.36 to be exact). The bigger the value of *τ*, the longer it takes for the number of experts to decrease to 0.36 of *η ^max^*. For example, if *η ^max^* is 20, a *τ* of two years means that, if two years pass without WCP use (or other complex practice), only seven individuals will be able to use the WCP, whereas a *τ* of eight years means that, it takes eight years without a use of the WCP for the number of experts to decline to seven.

As with the values of *θ* and *v*, the values of *τ* and *η ^max^* are constant among the temporal sequences that comprise a simulation but vary among simulations. We varied *η ^max^* between 3 and 60, and *τ* between 1 and 16 years. The minimum and maximum values for *η ^max^* are consistent with the ancient DNA-derived estimates of the size of Neanderthal groups ^24, 63, 64^. Assuming that the hypothetical Neanderthal group comprised 60 individuals (including elderly and children), we conceptualized values of *η ^max^* close to three as modeling the WCP as highly specialized knowledge and skills, and values of *η ^max^* close to 60 as modeling the WCP as widely distributed knowledge and skills. That everybody in the group was skilled in the WCP, even babies, is an extreme scenario, but is the most conservative consideration possible for the sake of the WCP retention (or other cultural practices) in our model.

The lower and upper limits of the range of values of *τ* were based on estimates of the rate of decay we derived from data presented in studies that have investigated the loss of procedural-motor skills ^39, 65– 68^. Procedural-motor skills, consisting of a set of well-established sequences that have to be repeated in a certain, specific order, like CRP, with a clear beginning and end, are the ones that best reflect the WCP due to the sequential nature of fire-caring, building camp-fires from embers and long term fire-curation, plus the right use of materials and kinds of tinder and woods ^8, 27, 30, 31^. We fitted exponential curves to the decay data reported in the studies and then inferred *τ* values. The estimated *τ* ranged from two months to four years (refer to extended data- Table S1 ^69^). We opted for a lower limit for *τ* of one year rather than two months because we were interested in the impact of variation in the intervals between uses of the WCP at the annual scale, plus the bigger the *τ,* the more conservative our analysis is. For the upper limit of *τ*, we selected 16 years because it allowed us to investigate the effect of slower decay rates than the maximum ones found in the literature for procedural-motor skills ^67^, also making our analysis more conservative.

### Deciding whether a group will use it or lose it

For all the temporal sequences generated with the numerical version of EMBERS, we used one person as the *Minimum Threshold* for the retention of the use of the WCP (or any other complex skill). We did so because, as we discussed earlier, the actions necessary to use wildfire to start a campfire in a temperate zone must be carried out in the right order. A corollary of this is that at least one person in the hypothetical Neanderthal group must know the right order of the actions for the group to be able to retain the ability to use the WCP. Thus, in the temporal sequences generated with the numerical version of EMBERS, the WCP was deemed permanently lost if the number of experts dropped below one.

Because the minimum threshold for the number of experts is one, it is possible to compute the maximum length of time a group can retain the WCP without using it. We refer to this variable as *Maximum Time* or Δ *t ^max^*. It is defined as the interval between the start of the exponential decay process described in the last section and *η*(Δ *t*) < 1. Therefore, we derive the expression for this interval as:

$$\text{Δ}t^{max}=\tau \;ln\;(\eta^{max}).\;(3)$$

In Figure 6, we illustrate the *Maximum Time* (interval between the origin and the magenta line, highlighted by black arrows) as the point where the number of experts drops below one. If the interval between uses exceeds *Maximum Time,* the number of experts drops below one. In other worlds, the *Minimum Threshold* links the *Maximum Number of Expert* ( *η ^max^*) and *Forgetting Time* ( *τ*) through *Maximum Time,* as depicted by Equation 3.

Interestingly, Δ *t ^max^* has a linear relationship with *Forgetting Time* ( *τ*) but a logarithmic relationship with *Maximum Number of Expert* ( *η ^max^*). The correlation plot presented in Panel A of Figure 7 sheds light on the relationship between *Forgetting Time* ( *τ*) and Δ *t ^max^*, as seen in Equation 3. It plots the values of Δ *t ^max^* that were obtained when Δ *t ^max^* was fixed at 12, against the corresponding values of *τ*. Correlation plots generated from other pairs of values of Δ *t ^max^* and *τ* show that Δ *t ^max^* is always linearly dependent on *τ*.

**Figure 8.**
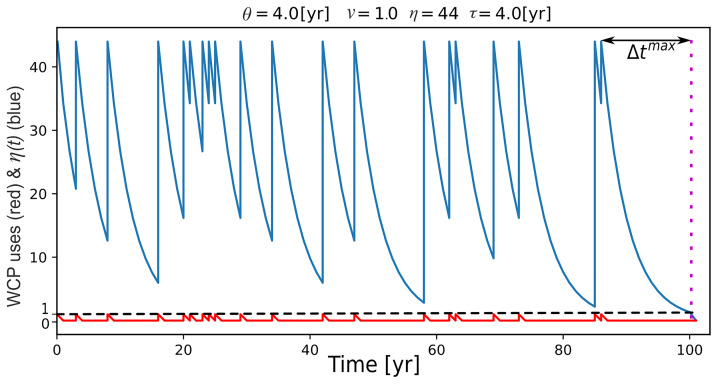
Dynamics of the process of the loss and recovery of experts. The number of experts is tracked by the blue line. The spikes in the red line at the bottom represent uses of the WCP (or any other complex practice). The threshold for retention of the ability to use the WCP (1 expert) is marked by the black dashed line towards the bottom of the panel. Starting at *t* = 0, the number of experts begins at *η ^max^*, decays exponentially until the WCP is used, and then recovers back to *η ^max^*. This process continues until the year 100, when *Use Interval* becomes larger than Δ *t ^max^* and *η(t)* drops below the group’s threshold for retention of the ability to use the WCP. At this point, the run comes to an end.

Given the foregoing, the recovery *vs* loss decision operation can be described in the following manner. Each temporal sequence begins with the number of experts at *η* (0) = *η ^max^*. This number starts to decay immediately. Whether or not the decay process continues depends on a use of the WCP occurring before the number of experts drops below 1, *i.e.*, *Δt*_1_ > Δ *t ^max^*. If this happens, the number of experts rapidly returns to *η ^max^* ( Figure 8). However, if the number of experts drops below one, the sequence ends and a permanent loss of the WCP is recorded. The process of decay and recovery repeats each *i* interval until either the number of experts drops below one ( Figure 8) or 1000 years has passed.

Formally, the recovery *vs* loss decision operation can be represented by the following expression:

$$\eta (t_{i}^{k})=\{\begin{array}{ll} \eta^{max} & if\;\eta (\text{Δ}t_{i}^{k})\;\geq \;1\\ 0 & if\;\eta (\text{Δ}t_{i}^{k})\;<\;1, \end{array}\;(4)$$

where is *η ^max^* the maximum number of experts for the simulation and *η*(Δ *t _i_*) is the number of group members capable of using the WCP at the end of a given interval (see Equation 1). The expression indicates that EMBERS calculates *η*( $\text{Δ}t_{i}^{k}$ ) for each $\text{Δ}t_{i}^{k}$ . If *N*(Δ *t _i_*) < 1, then the temporal sequence ends and a loss of the WCP (or any other complex practice) is recorded, $n_{l}^{k}$ = 1. However, if *η*( $\text{Δ}t_{i}^{k}$ ) ≥ 1, then *η*( ${\text{t}}_{\text{i}}^{\text{k}}$ ) recovers to *η ^max^*. If the temporal sequence reaches time $t_{i}^{k}$ = 1000, then $n_{l}^{k}$ = 0.

The loss *vs* retention operation can be summarized with the following expression:

$$n_{l}^{k}=\{\begin{array}{ll} 1 & if\;\eta (t_{i}^{k}=1000)\;=\;0\\ 0 & if\;\eta (t_{i}^{k}=1000)\;>\;1, \end{array}\;(5)$$

where $n_{l}^{k}$ counts for the number of losses *l* for each time series *k.* We opted to treat the recovery process as rapid partly because it is the simplest option but mainly because it reduces the probability of the WCP being lost, which meant that the analyses is the most conservative against loss, i.e. a stronger test of Sandgathe and colleagues ^13– 16^ hypothesis.

### Estimating the probability of loss for each simulation

The final operation of the numerical version of EMBERS estimates the probability of the hypothetical Neanderthal group losing the ability to use the WCP (or any other group keeping a complex practice) prior to 1000 years elapsing. The variability in the intervals that comprise a temporal sequence is important here. That the values of $\text{Δ}t_{i}^{k}$ for a temporal sequence will rarely be the same means there will be variability among the set of temporal sequences that comprise a simulation with respect to whether the use of the WCP is retained for 1000 years. This variability is used to estimate the probability of the loss of the WCP for a given simulation and therefore for the unique combination of values of the four key parameters ( *θ, v, η ^max^, τ*) employed in the simulation. For each simulation, EMBERS calculates *n _l_*, which is the number of temporal sequences experiencing a loss of the ability to use WCP before *L* = 1000. Formally *n _l_* can be defined as the sum of the $n_{l}^{k}$ values:


$${\text{n}}_{l}=\sum_{k=0}^{K}n_{l}^{k}.\;(6)$$

Thereafter, *n _l_* is divided by the total number of temporal sequences included in the simulation ( *K*). The resulting quotient is EMBERS’ estimate of the probability of retaining the WCP before *L* = 1000, for the simulation and, more importantly, for the unique combination of values of *θ*, *v*, *η ^max^*, and *τ* utilised in the simulation. Formally we can express this probability as:


$$P_{r}(\theta ,\nu ,\eta^{max},\tau )=1-n_{l}/K,\;(7)$$

where ( *θ, v, η ^max^, τ*) is the unique combination of *Use Interval* ( *θ*), *Variability* ( *v*), *Maximum Number of Experts* ( *η ^max^*), and *Forgetting Time* ( *τ*).

***Algorithm for the numerical version*.** Each simulation was based on a particular combination of values for the four key variables ( *θ, ν, η ^max^, τ*) and involved the creation of *K* independent temporal sequences ( *k* ∈ [0, *K*]). The simulations were run with the following algorithm:

1.    Compute a ( *θ*, [ *v* ⋅ *θ*] ^2^).

2.    Generate *n* $\text{Δ}t_{i}^{k}$ , *k* ∈ [0, K] sequences from the positive values of the distribution.

3.    For *k* ∈ [0, K] and for *i* ∈ [1, 2 *L*/ *θ*] do:

3.1 If $\text{Δ}t_{i}^{k}$ > Δ *t ^max^*, then the *k temporal sequence* is counted as a loss and ends, $n_{l}^{k}$ = 1, *k = k+1*.
3.2 If $\text{Δ}t_{i}^{k}$ < Δ *t ^max^*, then the *i* time increases as *t _i_* = *t*_*i*–1_ + Δ *t _i_*, *t*_0_ = 0.
3.3 If *t _i_* > *L*, then the series ends, $n_{l}^{k}$ = 0, *k = k+1*.

4.    When *k = K*, ${\text{n}}_{l}=\sum_{k=0}^{K}n_{l}^{k}$ , *P _r_* ( *θ, v, η ^max^,τ*) = 1 – *n _l_*/ *K*.

## The analytical version of EMBERS

In this section we outline the analytical version of EMBERS. This version represents a different solution to the problem of estimating the probability a group losing a complex practice in *L*, given different combinations of values of the four key variables, *θ, v, η ^max^*, and *τ*. Analytical solutions are only possible for some systems, but when they are feasible, they have the advantage of being quick to compute.

### Main parameters and operations

The analytical version of EMBERS employs the same values for *L* and the key variables as the numerical version of the model ( Table 1). It also employs the maximum time function utilized in the numerical version of EMBERS, Δ *t ^max^* (see Equation 3).

In computing the solution for each set of variable values we utilize the *Probability of Discontinuity* ( *P _d_*), which is the average probability that the group loses a complex practice after each use. Because the values for *Use Interval* are derived from a normal distribution ( Figure 5), we can easily compute *P _d_* with the aid of the error function (erf), which provides the probability that a value is bigger or smaller than a given threshold. In EMBERS, the threshold is the maximum time Δ *t ^max^*, and the distribution of values is given by ( *θ*, [ *v* · *θ*] ^2^). Therefore, the probability that any given interval is bigger than Δ *t ^max^* is:

$$P_{d}(N(\theta ,{\left[\theta \cdot \nu \right]}^{2}),\text{Δ}t^{max})=\frac{1}{2}\left[1-\text{erf}\left(\frac{\text{Δ}t^{max}-\theta }{\theta \cdot \nu \sqrt{2}}\right)\right].\;(8)$$

Next, we define the *Probability of Retention* ( *P _r_*), which is the probability that no interval between potential uses is longer than Δ *t ^max^* for the time length *L*. For simplicity, we assume that the number of intervals in *L* is the ratio between *L* and the mean *θ.* Given this, the probability that at least one of the intervals in *L* is longer than Δ *t ^max^* is simply 1 minus *P _d_* to the power of the number of intervals. Accordingly, *P _r_* is defined as: 


$$P_{r}(P_{d},L/\theta )=1-P_{d}^{L/\theta }.\;(9)$$

It is important to note that, because *P _d_* is the average discontinuity probability in each interval, the more intervals in *L*, the lower the probability of retention ( *P _r_*).

## Data Availability

All data are available in the main text or the supplementary materials. Supplementary materials can be found together with the code in the following: https://github.com/andreuandreu/EMBERS/tree/v3.2 Archived software and supplementary material available from Zenodo: andreuandreu/EMBERS: https://doi.org/10.5281/zenodo.15722063 ^69^ License: *European Union Public License 1.2*
