# Supplementary material for: Use it or lose it: A model-based assessment of the hypothesis that European Neanderthals relied on wildfires to create their campfires
Source: Open Res Eur. 2025 Nov 27;5:205. Originally published 2025 Jul 30. [Version 2] doi: 10.12688/openreseurope.20477.2 (PMC12775662; doi:10.12688/openreseurope.20477.2)
Supplement: Peer review history

# Reviewer report: Reviewer response for version 2

**DOI:** 10.21956/openreseurope.23775.r66416

**Published:** 2026-01-07

**Authors:** Gabriela Amorós

**Affiliations:** 1 University of Murcia, Murcia, Spain

This paper offers a thoughtful and timely reassessment of a long-standing assumption in Neanderthal research: that habitual fire use necessarily implies habitual fire-making. By explicitly modelling the ecological availability of wildfire under different climatic and vegetational scenarios, the authors move the debate away from technological dichotomies and toward a more realistic landscape-based perspective. In my view, this shift is both necessary and overdue.

 Recent developments increasingly emphasize that Neanderthal lifeways unfolded within highly heterogeneous landscapes, where vegetation structure, fuel continuity, and fire regimes would have varied substantially across space and time. In this broader context, the model presented here aligns well with recent landscape-scale and palaeoecological approaches highlighting the role of ecological thresholds and environmental constraints in shaping human fire use (Refer ref no.1).

 I find particularly thought-provoking  the paper’s implicit warning against asymmetric reasoning in the archaeological record. The absence of clear fire-making technologies is often treated as a deficit, whereas the systematic acquisition of fire from natural sources is seldom explored quantitatively. By demonstrating that repeated fire use could, under certain conditions, be sustained without habitual ignition, the authors provide a productive reframing that encourages greater interpretive caution and conceptual flexibility.

 This contribution does not seek to close the debate on Neanderthal fire use, but rather to reopen it on firmer ecological and theoretical grounds. As such, it represents a stimulating and welcome addition to ongoing discussions on Neanderthal adaptability and human–environment interactions.

 GABRIELA AMORÓS, University of Murcia

If any, are all the source data and materials underlying the results available?

Yes

Is the work original in terms of material and argument?

Yes

Is the work clearly and cogently presented?

Yes

Is the argument persuasive and supported by evidence?

Yes

Does the research article contribute to the cultural, historical, social understanding of the field?

Yes

Does it sufficiently engage with relevant methodologies and secondary literature on the topic?

Yes

Reviewer Expertise:

Ecosystems of Neanderthals, Palaeoecology, Palaeobotany, Paleoart, Visual Arts, Science Education

I confirm that I have read this submission and believe that I have an appropriate level of expertise to confirm that it is of an acceptable scientific standard.

## References

[1] : Beyond the cold steppes: Neanderthal landscapes and the neglect of flora. *Quaternary Science Reviews* .2026;371: 10.1016/j.quascirev.2025.109673 10.1016/j.quascirev.2025.109673

[2] : The Mismeasure of Neanderthals. *Earth History and Biodiversity* .2026;7: 10.1016/j.hisbio.2025.100036 10.1016/j.hisbio.2025.100036

# Reviewer report: Reviewer response for version 2

**DOI:** 10.21956/openreseurope.23775.r64936

**Published:** 2026-01-07

**Authors:** Michelle Scalise Sugiyama

**Affiliations:** 1 University of Oregon, Eugene, USA

I have no further comments.

If any, are all the source data and materials underlying the results available?

Yes

Is the work original in terms of material and argument?

Yes

Is the work clearly and cogently presented?

Yes

Is the argument persuasive and supported by evidence?

Yes

Does the research article contribute to the cultural, historical, social understanding of the field?

Yes

Does it sufficiently engage with relevant methodologies and secondary literature on the topic?

Partly

Reviewer Expertise:

The hunting-and-gathering ecological niche, including: cultural transmission and cumulative culture; evolution of teaching/learning; traditional ecological knowledge; evolution of symbolic behavior; oral tradition; evolution of play

I confirm that I have read this submission and believe that I have an appropriate level of expertise to confirm that it is of an acceptable scientific standard.

# Reviewer report: Reviewer response for version 2

**DOI:** 10.21956/openreseurope.23775.r65037

**Published:** 2026-01-06

**Authors:** José Luis Guil-Guerrero

**Affiliations:** 1 University of Almeria, Almería, Andalusia, Spain

Title:  Use it or lose it: A model-based assessment of the hypothesis that European Neanderthals relied on wildfires to create their campfires

 Journal: Open Research Europe

 # Global evaluation

 This article presents a sophisticated and carefully argued modelling assessment of the "wildfire hypothesis" for Neanderthal fire use. The EMBERS model is comprehensively delineated, internally consistent, and firmly rooted in ethnographic and cognitive literature. The study provides compelling evidence that the loss of wildfire-dependent fire-use knowledge is both plausible and likely under realistic conditions, thereby reinforcing the validity of Sandgathe et al.'s hypothesis. However, the work sometimes overstates the strength of inferences, conflates plausibility with explanatory sufficiency, and relies on assumptions that deserve tighter archaeological and ecological anchoring.

 # Conceptual issues

 -The central conceptual concern is the equation of the modelled cultural loss with the archaeological absence of fire. While the model shows that fire-use expertise may be lost despite occasional wildfire encounters, it does not directly demonstrate that such loss would necessarily produce the specific archaeological signatures observed. This issue is recognised, but not yet fully resolved (pp. 10–12).

 -Secondly, the Wildfire Cultural Package (WCP) is regarded as a cohesive, cumulative solution, where the loss is essentially binary (see pages 4 to 5 for further details). Components of fire use (e.g. ember transport, fuel selection, fire maintenance) may degrade asynchronously. This simplification may exaggerate the likelihood of total loss.

 -Thirdly, it is important to note that the assumption of group isolation (pp. 4–6, 12) is crucial to the results but is archaeologically weakly constrained. While genetic data are cited, social contact sufficient for ember exchange could occur without substantial gene flow.

 -Fourthly, the model assumes that memory decay is the primary loss mechanism. However, it should be noted that demographic shocks, seasonal mobility and ecological knowledge transmission may interact non-linearly with forgetting. These factors will be addressed in future work, but are central to the use of fire.

 # Issues by sections

 -Page 2 (Abstract & Results)

 It has been noted that the statement 'losing the ability to use wildfire was more likely than retaining it' risks misinterpretation. The model demonstrates the loss of expertise rather than the loss of fire use itself. This distinction should be clarified at the outset.

 -Page 4 (Model setup).

 The model only considers lightning-caused wildfires, but there are other natural ignition sources to consider, such as peat fires and human-mediated ember persistence. This simplification biases results towards loss without quantifying its effect.

 -Pages 5–6 (Assumptions on expert recovery).

 It is evident that the instantaneous reset of expert numbers after each successful use of the WCP is unrealistic and internally inconsistent with the emphasis on fragile transmission. This assumption strongly favours retention, yet this is not explored parametrically.

 -Pages 7–9, (Results)

 It is important to note that the repeated claim that loss is "more likely than retention" could be misread as a real-world frequency statement, rather than a statement conditional on parameter space sampling. It is important to reiterate the conditional nature of this probability.

 -Pages 10–11 (Critique of alternatives)

 The dismissal of economic and ephemeral-fire hypotheses relies heavily on the assumption that unused skills inevitably decay rapidly. While there may be some truth to this for certain procedural skills, it is more of an assertion than a demonstration when it comes to fire-making specifically.

 # Suggestions for improvement

 1. The authors should explicitly map model outcomes to archaeological expectations, clarifying what degree and duration of cultural loss would plausibly result in the observed reduction of fire proxies.

 2. The WCP should be partially decomposed, allowing for graded loss of fire-related competencies rather than an all-or-nothing threshold.

 3. Incorporating limited intergroup contact or ember exchange in sensitivity tests would substantially strengthen the realism of the conclusions.

 4. The paper would benefit from a clearer separation between plausibility testing and hypothesis validation. This would avoid any language that could be interpreted as confirming the wildfire hypothesis rather than bounding its feasibility.

If any, are all the source data and materials underlying the results available?

Yes

Is the work original in terms of material and argument?

Yes

Is the work clearly and cogently presented?

Yes

Is the argument persuasive and supported by evidence?

Partly

Does the research article contribute to the cultural, historical, social understanding of the field?

Yes

Does it sufficiently engage with relevant methodologies and secondary literature on the topic?

Yes

Reviewer Expertise:

Palaeonthropology, Food Technology, Wild Edible Plants

I confirm that I have read this submission and believe that I have an appropriate level of expertise to confirm that it is of an acceptable scientific standard, however I have significant reservations, as outlined above.

# Reviewer report: Reviewer response for version 1

**DOI:** 10.21956/openreseurope.22159.r57925

**Published:** 2025-11-08

**Authors:** Michelle Scalise Sugiyama

**Affiliations:** 1 University of Oregon, Eugene, USA

This important article calls out a serious shortcoming in the study of cumulative culture evolution: the lack of attention to memory decay and means of countering it. Consequently, little is known about the frequency with which knowledge decay occurred in past hominin environments or the conditions that contributed to it. To explore these questions, the article uses existing research on Neanderthal fire use. Oddly, several Middle Paleolithic Neanderthal sites exhibit a pronounced decrease in evidence of fire use during colder periods. A possible explanation for this decline is that Neanderthals were incapable of producing fire from scratch and instead relied on wildfire embers for ignition. Because lightning strikes and consequent wildfires are less frequent under cold, dry conditions, Neanderthals living during these periods would have had fewer opportunities to make fire, raising the intriguing possibility that this knowledge decayed during colder periods (and, presumably, was re-discovered during warmer periods when wildfires were more frequent).

 The authors use formal modeling to investigate this process, coding for four mechanisms of knowledge/skill loss: variability in use, interval between uses, memory decay, and number of experts. Two important takeaways from this effort are that (1) loss was more likely than retention and (2) many combinations of variability and use interval led to a total loss of fire-making knowledge over the 1000-year period modeled. In light of these findings, the authors stress the need for greater attention to memory decay and ways of countering it in cultural evolutionary research, claiming that they “have only been able to identify three recent relevant publications” (11).

 This claim raises two issues of concern. First, why limit the search to recent publications? In my own research, I have reaped insights from work published decades ago. For example, despite their flaws—or perhaps because of them--Bartlett’s (1932) experiments using “The War of the Ghosts” tale are highly instructive. Without a thorough search of the literature, the authors cannot be certain that memory decay and means of countering it have not been productively addressed elsewhere.

 This brings us to the second issue, which concerns the nature and scope of research on cumulative culture evolution. The authors are correct that evolutionary scholars (i.e., scientists who study hominin or hominid evolution) have largely ignored an important component of cultural transmission: the media used by oral cultures to encode and transmit knowledge, and the mechanisms and strategies used to prevent knowledge decay and corruption. Their critique of this omission is valid, as is their call for “researchers interested in cultural evolution to increase the number of studies dealing with … ways of retaining knowledge and skills” (11). However, their conceptualization of “relevant publications” appears to be extremely narrow—so narrow that it excludes recent research that expressly addresses this issue (e.g., Nowell 2021; Scalise Sugiyama 2024; Scalise Sugiyama & Reilly 2023). Moreover, the authors do not follow through on their critique by directing readers to the vast body of research on the challenges posed by oral transmission of communal knowledge and/or strategies used to surmount them (e.g., Hagar 1900; Lord 1960; Berlin & O’Neill 1981; Ong 1982; Minc 1986; Cruikshank 1990; Rubin 1995; Basso 1996; Johnson 1998; Sobel & Bettles 2000; Barber & Barber 2004; Ludwin et al. 2007; Kelly 2015; Clarke 2018; Curran et al. 2019; Scalise Sugiyama 2021, 2022). A related body of research examines the role of play in the entrainment and maintenance of physical skills (e.g., Bock & Johnson 2004; Petersen 2004; Scalise Sugiyama et al. 2018). While most of this research is not “cultural evolutionary theory” in the strict sense used by the authors, it is most certainly research on “ways of countering memory decay” and “the maintenance of existing cultural traits” (11). This work is the logical starting place for cultural evolutionary theorists seeking to develop models that incorporate the mechanics of cultural loss and means used to prevent it.

 These omissions are all the more perplexing given that one of the co-authors, Riede, has published several articles on the role of play in cultural evolution, including the use of toys for teaching. One of these studies, which examines the cross-cultural pervasiveness of string figures in hunter-gatherer societies, argues that “games and pastimes like string figures *allow individuals to freely practice manual dexterity and cognitive skills*” (Kaaronen et al. 2024:9; my emphasis). In light of the present paper’s “use it or lose it” argument, the failure to mention play as a means of countering knowledge/skill loss is a missed opportunity.  

 In their discussion of directions for future research, the authors note that their model may actually have *overestimated* the probability of retention of pyrotechnical knowledge because it did not factor in the possibility of acquiring embers from neighboring groups. I encourage the authors to pursue this question in future research, as in my own work I have encountered numerous references to the borrowing (or acquisition) of fire from neighbors or other villages. Significantly, this information is often found in stories, which are widely recognized as an important means of preserving and transmitting accumulated communal knowledge in hunter-gatherer and other oral cultures. The following passage from a Wasco tale illustrates this practice: 

 Towards midnight of the following day the fire went out, and in the village the fires went out in every house. Next day the father said to the eldest boy, “Go over to Tenino [a neighboring village] and get fire.” The two boys started. Towards sundown they reached Tenino …. Their father had made a stick of cedar-bark for them with little cracks in it, good to hold fire; they crept up to the fire and lighted this stick. (Sapir 1909:243)

 As this example indicates, the mechanisms used by hunter-gatherers to maintain existing cultural traits are right under our noses, and are well-documented ethnographically. To detect them, cultural evolutionary theorists need to broaden their search parameters.

 References

 Barber, E. J. W. & Barber, P. (2004). *When they severed earth from sky: How the human mind shapes myth*. Princeton University Press. https://ebookcentral.proquest.com/lib/uoregon/detail.action?docID=827791

 Bartlett, F. C. (1932). *Remembering*. Cambridge University Press. https://pure.mpg.de/rest/items/item_2273030/component/file_2309291/content 

 Basso, K. (1996). *Wisdom sits in places: Landscape and language among the Western Apache*. University of New Mexico Press.

 Berlin, B. & O’Neill, J. P. (1981). The pervasiveness of onomatopoeia in Aguaruna and Huambisa bird names. *Journal of Ethnobiology*, *1*(2), 238-261. https://ethnobiology.org/sites/default/files/pdfs/JoE/1-2/Berlin_ONeill1981.pdf

 Bock, J. & Johnson, S. E. (2004). Subsistence ecology and play among the Okavango Delta peoples of Botswana. *Human Nature, 15*(1), 63-81.

 Clarke, P. A. (2018). The Ngarrindjeri nomenclature of birds in the Lower Murray River region, South Australia. *Transactions of the Royal Society of South Australia*, 1-29.

 Cruikshank, J. (1990). Getting the words right: Perspectives on naming and places in Athapaskan oral history. *Arctic Anthropology, 27*(1), 52-65. https://www.jstor.org/stable/40316196

 Curran, G., Barwick, L., Turpin, M., Walsh, F. & Laughren, M. (2019). Central Australian Aboriginal songs and biocultural knowledge: Evidence from women’s ceremonies relating to edible seeds. *Journal of Ethnobiology, 39*(3), 354-370.

 Hagar, S. (1900). The celestial bear. *The Journal of American Folklore*, *13*(49), 92-103. https://doi.org/10.2307/533799

 Johnson, D. (1998). *Night Skies of Aboriginal Australia: A Noctuary*. Sydney University Press. http://www.aboriginalastronomy.com.au/wp-content/uploads/2018/05/Night_Skies.pdf

 Kaaronen, R. O., Walsh, M. J., Henrich, A. K., Wisher, I., Miu, E., Manninen, M. A., ... & Riede, F. (2024). A global cross-cultural analysis of string figures reveals evidence of deep transmission and innovation. *Journal of the Royal Society Interface*, *21*(221), 1-15.

 Kelly, L. (2015). *Knowledge and Power in Prehistoric Societies: Orality, Memory, and the Transmission of Culture*. Cambridge University Press.

 Lord, A. (1960). *The Singer of Tales*. Harvard University Press.

 Ludwin, R.S., Smits, G.J., Carver, D., James, K., Jonientz-Trisler, C., McMillan, A.D., Losey, R., Dennis, R., Rasmussen, J., De Los Angeles, A. & Buerge, D. (2007). Folklore and earthquakes: Native American oral traditions from Cascadia compared with written traditions from Japan. *Geological Society, London, Special Publications*, *273*(1), 67-94.

 Minc, L. (1986). Scarcity and survival: the role of oral tradition in mediating subsistence crises. *Journal of Anthropological Archaeology,* *5*(1), 39-113.

 Nowell, A. (2021). *Growing Up in the Ice Age*. Oxbow. https://ebookcentral.proquest.com/lib/uoregon/detail.action?docID=30542735

 Ong, W. J. (1982). *Orality and Literacy*. Routledge.

 Petersen, H. C. (2004). Kayak sports and exercises. In J. D. Heath & E. Arima (Eds.), *Eastern Arctic Kayaks: History, Design, Technique*, 99-102. University of Alaska Press.

 Rubin, D. (1995). *Memory in oral traditions: The cognitive psychology of epic, ballads, and counting-out rhymes*. Oxford University Press.

 Sapir, E. (1909). *Wishram Texts, Together with Wasco Tales and Myths*. Late E.J. Brill.

 Scalise Sugiyama, M. (2021). Co-occurrence of ostensive communication and generalizable knowledge in forager storytelling: Cross-cultural evidence of teaching in forager societies. *Human Nature, 32*(1), 279-300.

 Scalise Sugiyama, M. (2022). Animal story songs: A hunter-gatherer pedagogical strategy. In J. Friedmann (Ed.), *Music in Human Experience: Interdisciplinary Perspectives on a Musical Species,* 183-205. Cambridge Scholars.

 Scalise Sugiyama, M. (2024). Oral storytelling: humanity's first data management system? *Evolution and Human Behavior*, *45*(3), 241-251.

 Scalise Sugiyama, M., Mendoza, M., Sugiyama, L. & White, F. (2018). Coalitional play fighting and the evolution of coalitional intergroup aggression. *Human Nature*, *29*(3), 219–244.

 Sobel, E. & Bettles, G. (2000). Winter hunger, winter myths: subsistence risk and mythology among the Klamath and Modoc. *Journal of Anthropological Archaeology,* *19*(3), 276-316.

If any, are all the source data and materials underlying the results available?

Yes

Is the work original in terms of material and argument?

Yes

Is the work clearly and cogently presented?

Yes

Is the argument persuasive and supported by evidence?

Yes

Does the research article contribute to the cultural, historical, social understanding of the field?

Yes

Does it sufficiently engage with relevant methodologies and secondary literature on the topic?

Partly

Reviewer Expertise:

Hunter-gatherer ecology and cultural adaptation, including: cultural transmission and cumulative culture; teaching/learning; traditional ecological knowledge; evolution of symbolic behavior; oral tradition; evolution of play

I confirm that I have read this submission and believe that I have an appropriate level of expertise to confirm that it is of an acceptable scientific standard, however I have significant reservations, as outlined above.

## References

[1] : Wisdom sits in places: Landscape and language among the Western Apache. *University of New Mexico Press* .1932;

[2] : The pervasiveness of onomatopoeia in Aguaruna and Huambisa bird names. *Journal of Ethnobiology Press* .1981;

[3] : Subsistence ecology and play among the okavango delta peoples of botswana. *Human Nature* .2004;15(1) : 10.1007/s12110-004-1004-x 63-81 10.1007/s12110-004-1004-x 26190294

[4] : The Ngarrindjeri nomenclature of birds in the Lower Murray River region, South Australia. *Transactions of the Royal Society of South Australia* .2019;143(1) : 10.1080/03721426.2018.1534530 118-146 10.1080/03721426.2018.1534530

[5] : Getting the words right: Perspectives on naming and places in Athapaskan oral history. *Arctic Anthropology, 27(1), 52-65* .1990; Reference source

[6] : Central Australian Aboriginal Songs and Biocultural Knowledge: Evidence from Women's Ceremonies Relating to Edible Seeds. *Journal of Ethnobiology* .2019;39(3) : 10.2993/0278-0771-39.3.354 10.2993/0278-0771-39.3.354

[7] : A global cross-cultural analysis of string figures reveals evidence of deep transmission and innovation. *Journal of The Royal Society Interface* .2024;21(221) : 10.1098/rsif.2024.0673 10.1098/rsif.2024.0673 PMC1161452839626748

[8] : Knowledge and Power in Prehistoric Societies.2015; 10.1017/CBO9781107444973 10.1017/CBO9781107444973

[9] : Folklore and earthquakes: Native American oral traditions from Cascadia compared with written traditions from Japan. *Geological Society, London, Special Publications* .2007;273(1) : 10.1144/GSL.SP.2007.273.01.07 67-94 10.1144/GSL.SP.2007.273.01.07

[10] : Scarcity and survival: The role of oral tradition in mediating subsistence crises. *Journal of Anthropological Archaeology* .1986;5(1) : 10.1016/0278-4165(86)90010-3 39-113 10.1016/0278-4165(86)90010-3

[11] : Memory In Oral Traditions.1995; 10.1093/oso/9780195082111.001.0001 10.1093/oso/9780195082111.001.0001

[12] : Publications of the American Ethnological Society: Volume I. Fox Texts. *The Journal of American Folklore* .1909;22(85) : 10.2307/534756 10.2307/534756

[13] : Co‐occurrence of Ostensive Communication and Generalizable Knowledge in Forager Storytelling. *Human Nature* .2021;32(1) : 10.1007/s12110-021-09385-w 279-300 10.1007/s12110-021-09385-w 33844163

[14] : Oral storytelling: humanity's first data management system?. *Evolution and Human Behavior* .2024;45(3) : 10.1016/j.evolhumbehav.2024.03.002 241-251 10.1016/j.evolhumbehav.2024.03.002

[15] : Coalitional Play Fighting and the Evolution of Coalitional Intergroup Aggression. *Human Nature* .2018;29(3) : 10.1007/s12110-018-9319-1 219-244 10.1007/s12110-018-9319-1 29959606

[16] : Cross-cultural forager myth transmission rules: Implications for the emergence of cumulative culture. *Evolution and Human Behavior* .2023;44(6) : 10.1016/j.evolhumbehav.2023.01.012 566-583 10.1016/j.evolhumbehav.2023.01.012

[17] : Winter Hunger, Winter Myths: Subsistence Risk and Mythology among the Klamath and Modoc. *Journal of Anthropological Archaeology* .2000;19(3) : 10.1006/jaar.2000.0365 276-316 10.1006/jaar.2000.0365

[18] : When they severed earth from sky: How the human mind shapes myth. *Princeton University Press.* .2004; Reference source

[19] : Remembering. *Cambridge University Press* .1932; Reference source

[20] : The Celestial Bear. *The Journal of American Folklore* .1900;13(49) : 10.2307/533799 10.2307/533799

# Response

**Published:** 2025-11-21

**Authors:** Andreu Arinyo i Prats

**Affiliations:** Arkæologi og Kulturarvsstudier, Aarhus Universitet, Aarhus, Central Denmark Region, Denmark

**Response to Reviewer 2 (Prof. Michelle Scalise Sugiyama)**   We thank Reviewer 2 for her thoughtful review. We appreciate the time and care she devoted to engaging with our paper. Below we provide responses to the main points she raised.  

**1. Focus on recent publications**  

***1.1. Reviewer comment*** The reviewer questions why our literature review was restricted to recent publications.  

***Response:*** We suspect our phrasing may have caused some confusion here. Our intention was not to disregard earlier scholarship. Rather, our goal was to highlight the fact that *within the field of cultural evolution studies*, there has been limited recent engagement with the issue of cultural loss in the last few decades. We have tried to clarify this point in the revised version of the Discussion. The start of the relevant paragraph now reads as follows:   “The present study also has implications for the ongoing effort to develop an adequate theory of cultural evolution ^43^ . In the last 25 years, researchers working in the field of cultural evolutionary studies have discussed cultural loss, but they have done so primarily in the context of trying to elucidate the relationship between cultural complexity and demography (e.g., 44–53). Little attention has been paid to the importance of cultural loss relative to cumulative cultural evolution, or to the specific mechanics of cultural loss”   We believe the revised section better communicates our intent.   Before moving on to the next point, it is worth noting that elsewhere in the paper we do reference a wide range of earlier and foundational work — starting with Rivers (1912) and including little-known studies focused on CPR and military skills, from the 1970s and 1980s.  

**2. Overly narrow scope of the cited literature**  

***2.1. Reviewer comment*** The reviewer suggests that our conceptualization of relevant publications is too narrow and results in the exclusion of several pertinent works on oral transmission, communal knowledge, and strategies for preventing memory decay.  

***Response:*** We agree that many of the studies cited by the reviewer offer valuable insights into the way by oral cultures preserve and transmit knowledge. We omitted them for the sake of brevity but accept that we went too far. To rectify the situation, we have amended the fourteenth paragraph of the Discussion. Its final sentence now directs the reader to studies dealing with memory loss and retention in other fields of study. The sentence in question reads as follows:   As part of this effort, it would be sensible to tap into the work being carried out by researchers interested in cultural preservation (Chaplin-Kramer *et al.*, 2022; Aguilar and Webb, 2024; Arinyo-i-Prats, Turner and Latosky, 2025), the role of oral traditions in cultural maintenance (Scalise Sugiyama, 2021, 2024; Scalise Sugiyama and Reilly, 2023), children’s play and oral storytelling in small-scale societies (Scalise Sugiyama, 2017; Langley, 2018; Riede *et al.*, 2018; Nowell, 2023), and the role of memory in human affairs (Arthur and Day, 2019; Corbett *et al.*, 2020; Kusumastuti *et al.*, 2022; Olivier, 2024). Some of the recent work on culture in non-human animals is also likely to be helpful (Brakes *et al.*, 2019; van Dooren *et al.*, 2024; Oestreich, Barlow and Hersh, 2025).  

**3. Role of play in maintaining skills and knowledge**  

***3.1. Reviewer comment*** The reviewer highlights the omission of research on the role of play in skill acquisition and maintenance, noting its relevance to our “use it or lose it” framework and citing examples such as Bock and Johnson (2004), Petersen (2004), and Scalise Sugiyama et al. (2018). The reviewer also points out that one of us has published on this very topic.  

***Response:*** We appreciate this observation. We are indeed aware of the importance of play in cultural transmission and skill retention and have previously published on related themes. However, our search of the ethnographic literature found little direct evidence for children’s participation in fire foraging, ember transport, or fire maintenance — the specific focus of our study. For this reason, we chose not to expand on the topic in depth to preserve the paper’s focus. Nevertheless, we have suggested that cultural evolutionary theorists should engage with the work on children’s play, at the end of the fourteenth paragraph of the Discussion.  

**4. Misinterpretation of model limitations regarding ember acquisition**  

***4.1. Reviewer comment*** Reviewer 2 notes that we argued that our model may have *overestimated* the probability of pyrotechnical knowledge retention because it did not factor in ember acquisition from neighbouring groups. She encourages us to explore this possibility further.  

***Response:*** As we explain towards the end of the Discussion, we intend to explore this issue in a future modeling study. The problem is that our decision not to factor in the possibility of ember acquisition from neighboring groups is potentially counterbalanced by our decision not to model the loss of experts via death and migration. While the former can be expected to have led us to overestimate the probability of loss of the WCP, the latter can be expected to have led us to underestimate the probability of loss of the WCP. However, because the dynamics of these processes are likely to be complex, we cannot say anything more concrete about their probable impact on the results of the study at this stage. Elucidating the impact of the two variables on the probability of loss of the WCP will require additional, dedicated modeling work.        

**5. General remarks and integration of suggested literature**  

***5.1. Reviewer comment*** Reviewer 2 provides a helpful list of literature on oral transmission, skill retention, and memory maintenance, noting that our discussion could benefit from engaging with this body of work.  

***Response:*** We agree that citing more papers on oral transmission, skill retention, and memory maintenance would have been desirable, but we were keen to keep the text concise and focused. We still are, so we haven’t added all of the literature highlighted by the reviewer. Instead, we have selected what we consider to be the most relevant of the studies for inclusion.  

**6. Concluding remarks**

 Once again, we thank Reviewer 2 for her review. Her comments helped us improve the clarity, scope, and precision of our manuscript. We think the revised version now more accurately conveys our intended arguments and better acknowledges previous work. We believe the paper is now ready for the final stage of the publication process and hope that Reviewer 2 agrees.

# Reviewer report: Reviewer response for version 1

**DOI:** 10.21956/openreseurope.22159.r57927

**Published:** 2025-08-30

**Authors:** Michael Chazan

**Affiliations:** 1 University of Toronto, Toronto, Ontario, Canada

Arinyo-i-Prats et al. provide a welcome contribution to the argument that obligate cooking is an emergent aspect of genus Homo rather than a precondition for the evolution of a hominin with increased brain size (see discussion in Chazan 2017).  This article builds on the observation by Sandgathe and colleagues that in French Middle Paleolithic sites with a deep stratigraphic sequence there are fluctuations in the intensity of the signal for the use of fire as reflected in the frequency of burnt lithics.  Moreover, it is precisely in periods of colder climate conditions, when one would assume fire would be most useful, that this signal is weakest.  Sandgathe proposes that this pattern reflects the reliance of Neanderthal on natural fires, which are less frequent in cold periods due to a decrease in the frequency of lightening strikes.  Arinyo-i-Prats carry out a simulation to see whether loss of technical knowledge could account for this pattern.  Not surprisingly they find that there would be loss of technical knowledge over time that could lead to a loss of acquired skill.  This simulation brings attention to the social aspect of the use of fire that is often neglected in discussions that focus on diet and energetics.

 I can offer two comments on this discussion.  The first is that there is a lack of clarity in the authors’ use of nomenclature about the type of engagement with fire under consideration.  This is often described as ‘use of wildfire’ whereas the topic of this article is actually the maintenance of fire and the tending of fire in a way that facilitates cooking and other aspects of pyrotechnology.  The evidence from our research at Wonderwerk Cave demonstrates that the use of wildfire dates back at least to the Acheulean, but there is an important distinction between the technical knowledge needed to collect wildfire as opposed to the complex operations needed to maintain fire (Berna et al. 2012).  The maintenance of fire is not clearly evident at Wonderwerk or other sites with Acheulean evidence for the use of fire. 

 The second point stems from the type of model adopted for the transmission of knowledge found in this article.  Transmission of knowledge is seen in this article as the passing along of packets of information and seems to occur in a world devoid of other technologies and social relations.  It would be interesting to see the authors expand the scope of their work to include stone tools. In the Middle Paleolithic we often see relatively minor variation in lithic technology which speaks not only to transmission of skill and knowledge but also on the way stone tools (and this would be even more true of fire) are deeply embedded in social life and other technical domains, particularly food preparation.  What is lost in Arinyo-i-Prats et al.’s discussion of knowledge transmission are the traditions that constrain the choice of technical strategies.  A shift in the use of fire would require a very considerable loosening of cultural norms of behavior.  The ‘forgetting’ the authors describe would require a reorganization of food preparation and elements of social structure that would have been quite profound.  This aspect of the integration of technical and social aspects of hominin adaptation is somewhat lost by the kind of modeling presented here.  Nonetheless, the authors do succeed in shifting our focus in a productive manner and in pointing to areas of further research.  This work builds constructively on the observations by Sandgathe and colleagues make a strong case that not only was obligate cooking not characteristic of the earliest members of our genus, it was not even essential to the adaptations of Neanderthals, whose cranial capacity overlaps with that of modern humans.

If any, are all the source data and materials underlying the results available?

Yes

Is the work original in terms of material and argument?

Yes

Is the work clearly and cogently presented?

Yes

Is the argument persuasive and supported by evidence?

Partly

Does the research article contribute to the cultural, historical, social understanding of the field?

Yes

Does it sufficiently engage with relevant methodologies and secondary literature on the topic?

Yes

Reviewer Expertise:

Archaeology and human evolution. I do not have expertise in the technical aspects of the simulation presented in this article.

I confirm that I have read this submission and believe that I have an appropriate level of expertise to confirm that it is of an acceptable scientific standard.

## References

[1] : Toward a Long Prehistory of Fire. *Current Anthropology* .2017;58(S16) : 10.1086/691988 S351-S359 10.1086/691988

[2] : Microstratigraphic evidence of in situ fire in the Acheulean strata of Wonderwerk Cave, Northern Cape province, South Africa. *Proceedings of the National Academy of Sciences* .2012;109(20) : 10.1073/pnas.1117620109 10.1073/pnas.1117620109 PMC335666522474385

# Response

**Published:** 2025-11-21

**Authors:** Andreu Arinyo i Prats

**Affiliations:** Arkæologi og Kulturarvsstudier, Aarhus Universitet, Aarhus, Central Denmark Region, Denmark

**Response to Reviewer 1 (Prof. Michael Chazan)**   We thank Reviewer 1 for the time and care he devoted to reviewing our paper. Below we respond to his two points of critique.   

**1. Nomenclature and scope of the Wildfire Cultural Package**

***1.1. Reviewer 1 comment*** “The first is that there is a lack of clarity in the authors’ use of nomenclature about the type of engagement with fire under consideration. This is often described as ‘use of wildfire’ whereas the topic of this article is actually the maintenance of fire and the tending of fire in a way that facilitates cooking and other aspects of pyrotechnology. The evidence from our research at Wonderwerk Cave demonstrates that the use of wildfire dates back at least to the Acheulean, but there is an important distinction between the technical knowledge needed to collect wildfire as opposed to the complex operations needed to maintain fire (Berna et al. 2012). The maintenance of fire is not clearly evident at Wonderwerk or other sites with Acheulean evidence for the use of fire.”  

***Response:*** There appears to be a misunderstanding here. As we explain in the section of the paper titled “Key assumptions of the model”, we modeled the impact of several variables on the retention of the Wildfire Cultural Package or WCP. We did not conceptualize the WCP as limited to “the maintenance of fire and the tending of fires in a way that facilitates cooking and other aspects of pyrotechnology”. Rather, we conceptualized it as *all* the knowledge and skills that a Neanderthal group would have needed to collect and use wildfire. In the paper, we highlighted several of the actions that are necessary to use wildfire to start a campfire in a temperate zone, but this was not supposed to be an exhaustive list, which is why we prefaced the list with “[t]hese actions include”. That said, we accept it is possible that, as Prof. Chazan suggests, “there is an important distinction between the technical knowledge needed to collect wildfire as opposed to the complex operations needed to maintain fire”, and we will consider subdividing the WCP and modelling the elements of the package separately in a future extension of the present study. A key problem we will have to overcome, we suspect, is grounding the parameters for each of the elements of the package in the empirical literature.  

**2. Impact of loss of fire on other cultural traits**

***2.1. Reviewer 1 comment*** “The second point stems from the type of model adopted for the transmission of knowledge found in this article. Transmission of knowledge is seen in this article as the passing along of packets of information and seems to occur in a world devoid of other technologies and social relations. It would be interesting to see the authors expand the scope of their work to include stone tools. In the Middle Paleolithic we often see relatively minor variation in lithic technology which speaks not only to transmission of skill and knowledge but also on the way stone tools (and this would be even more true of fire) are deeply embedded in social life and other technical domains, particularly food preparation. What is lost in Arinyo-i-Prats et al.’s discussion of knowledge transmission are the traditions that constrain the choice of technical strategies. A shift in the use of fire would require a very considerable loosening of cultural norms of behavior. The ‘forgetting’ the authors describe would require a reorganization of food preparation and elements of social structure that would have been quite profound. This aspect of the integration of technical and social aspects of hominin adaptation is somewhat lost by the kind of modeling presented here.”    

***Response:*** This is an interesting point, one that deserves to be explored in detail in a future study. We share Prof. Chazan intuition but we’re aware of empirical studies that suggest that situation may be more complicated than he and we imagine. To address this point, we have added the following paragraph to the future directions section of the Discussion:   “ Another possibility for future research was suggested by Prof. Michael Chazan in his review of the present paper. Prof. Chazan suggested that we should consider the impact of the loss of the WCP on other dimensions of Neanderthal culture, especially their stone tools. The idea here is that the WCP would have been deeply integrated with other cultural behaviours and therefore its disappearance via forgetting would have resulted in reorganization of other parts of the Neanderthal cultural repertoire. We share this intuition and agree that it would be interesting to investigate whether the decline in fire evidence at the relevant Neanderthal sites is associated with changes in archaeological evidence that can be plausibly linked to subsistence or thermoregulation, such as the extent to which animal bones were processed to extract grease and the frequency of the remains of fur-bearing animal (see Collard *et al.*, 2016). However, it is worth noting that studies of cultural transmission in contemporary societies imply that the degree of integration and packaging among cultural traits is context-dependent (Cavalli-Sforza *et al.*, 1982; Guglielmino *et al.*, 1995; Jordan, 2014).  So, a failure to find a correlated change in, say, the extent to which animal bones were processed to extract grease would not necessarily be surprising, nor shall it be ruled out without proper study.    

**3. Concluding remarks**

 Once again, we thank Reviewer 1 for his comments, which we found both stimulating and helpful. We believe the paper is now ready for the final stage of the indexing process and hope that Reviewer 1 agrees
